# Prediction of incident heart failure in established atherosclerotic cardiovascular disease: the SMART2-HF model

**DOI:** 10.1093/eurheartj/ehag153

**Published:** 2026-03-11

**Authors:** Tessa H Reitsma, Carl-Emil Lim, Mari N Gynnild, Stephen Kaptoge, Laura Lõo, Joris Holtrop, Łukasz Kuźma, Lisa Pennells, Nathalie Conrad, Peter Ueda, Tomas Jernberg, Anna Kurasz, Spencer J Keene, Chimweta Chilala, Salil V Deo, Taavi Tillmann, Kamlesh Khunti, Massimo Piepoli, Xavier Rossello, Gianluigi Savarese, Håvard Dalen, Ph Gabriel Steg, Angela M Wood, Jennifer S Lees, Maryam Kavousi, John William McEvoy, Rudolf A de Boer, Charlotte Andersson, Deepak L Bhatt, Frank L J Visseren, Jannick A N Dorresteijn, Stefan Koudstaal, Emanuele Di Angelantonio, Steven H J Hageman, UCC-SMART study group & the ESC Cardiovascular Risk Collaboration, S I M Bongers, J L P van Giele, M Van Daal, M J Cramer, M G van der Meer, P van der Harst, H M Nathoe, J van Setten, M Teraa, N C Onland-Moret, M van Smeden, M H Emmelot-Vonk, P A de Jong, A T Lely, S Haitjema, M M Mokhles, Y M Ruigrok, M C Verhaar, J A N Dorresteijn, F L J Visseren

**Affiliations:** Department of Vascular Medicine and Endocrinology, University Medical Centre Utrecht, Utrecht 3508 GA, The Netherlands; Department of Medicine, Clinical Epidemiology Division, Karolinska Institutet, Stockholm, Sweden; Clinic of Cardiology, St.Olav University Hospital, Trondheim, Norway; Department of Circulation and Medical Imaging, Faculty of Medicine and Health Science, NTNU–Norwegian University of Science and Technology, Trondheim, Norway; K.G. Jebsen Center for Cardiac Biomarkers, Institute of Clinical Medicine, University of Oslo, Norway; Department of Public Health and Primary Care, British Heart Foundation Cardiovascular Epidemiology Unit, University of Cambridge, Papworth Road, Cambridge Biomedical Campus, Cambridge, UK; Victor Phillip Dahdaleh Heart and Lung Research Institute, University of Cambridge, Cambridge, UK; Institute of Family Medicine and Public Health, University of Tartu, Tartu, Estonia; Department of Vascular Medicine and Endocrinology, University Medical Centre Utrecht, Utrecht 3508 GA, The Netherlands; Department of Invasive Cardiology, Medical University of Białystok, Białystok, Poland; Department of Cardiac Surgery and Transplantology, National Medical Institute of the Ministry of Interior, Warsaw, Poland; Liverpool Centre for Cardiovascular Science, University of Liverpool, Liverpool, UK; Department of Public Health and Primary Care, British Heart Foundation Cardiovascular Epidemiology Unit, University of Cambridge, Papworth Road, Cambridge Biomedical Campus, Cambridge, UK; Victor Phillip Dahdaleh Heart and Lung Research Institute, University of Cambridge, Cambridge, UK; British Heart Foundation Centre of Research Excellence, University of Cambridge, Cambridge, UK; Nuffield Department of Women’s and Reproductive Health, University of Oxford, Oxford, UK; Department of Cardiovascular Sciences, KU Leuven, Leuven, Belgium; Department of Medicine, Clinical Epidemiology Division, Karolinska Institutet, Stockholm, Sweden; Department of Clinical Sciences, Cardiology, Karolinska Institutet Danderyd Hospital, Stockholm, Sweden; Department of Invasive Cardiology, Medical University of Białystok, Białystok, Poland; Department of Public Health and Primary Care, British Heart Foundation Cardiovascular Epidemiology Unit, University of Cambridge, Papworth Road, Cambridge Biomedical Campus, Cambridge, UK; Victor Phillip Dahdaleh Heart and Lung Research Institute, University of Cambridge, Cambridge, UK; Department of Public Health and Primary Care, British Heart Foundation Cardiovascular Epidemiology Unit, University of Cambridge, Papworth Road, Cambridge Biomedical Campus, Cambridge, UK; Victor Phillip Dahdaleh Heart and Lung Research Institute, University of Cambridge, Cambridge, UK; Surgical Services, Louis Stokes Cleveland VA Medical Center, Cleveland, OH, USA; Case School of Medicine, Case Western Reserve University School of Medicine, Cleveland, OH, USA; School of Health and Wellbeing, University of Glasgow, Glasgow,United Kingdom; Institute of Family Medicine and Public Health, University of Tartu, Tartu, Estonia; Diabetes Research Centre, Leicester General Hospital, University of Leicester, Leicester, UK; Department of Biomedical Science for Health, University of Milan, Milan, Italy; Clinical University Cardiology, IRCCS Policlinico San Donato, Milan, Italy; Hospital Universitario Son Espases, Health Research Institute of the Balearic Islands, Universitat Illes Balears, Palma de Mallorca, Spain; Department of Clinical Science and Education, Södersjukhuset; Karolinska Institute, Stockholm, Sweden; Clinic of Cardiology, St.Olav University Hospital, Trondheim, Norway; Department of Circulation and Medical Imaging, Faculty of Medicine and Health Science, NTNU–Norwegian University of Science and Technology, Trondheim, Norway; Levanger Hospital, Nord-Trøndelag Hospital Trust, Levanger, Norway; FACT, French Alliance for Cardiovascular Trials, AP-HP, Hôpital Bichat, INSERM U-1148, Université Paris-Cité, Paris, France; Department of Public Health and Primary Care, British Heart Foundation Cardiovascular Epidemiology Unit, University of Cambridge, Papworth Road, Cambridge Biomedical Campus, Cambridge, UK; Victor Phillip Dahdaleh Heart and Lung Research Institute, University of Cambridge, Cambridge, UK; National Institute for Health and Care Research Blood and Transplant Research Unit in Donor Health and Behaviour, University of Cambridge, Cambridge, UK; Health Data Research UK Cambridge, Wellcome Genome Campus and University of Cambridge, Cambridge, UK; British Heart Foundation Data Science Centre, Health Data Research UK, London, UK; School of Cardiovascular & Metabolic Health, University of Glasgow, Glasgow, UK; Department of Epidemiology, Erasmus MC, University Medical Centre Rotterdam, Rotterdam, The Netherlands; National Institute for Prevention and Cardiovascular Health, University of Galway School of Medicine, Galway, Ireland; Department of Cardiology, Thorax Center, Cardiovascular Institute, Erasmus MC, Rotterdam, The Netherlands; Department of Cardiology, Copenhagen University Hospital Herlev and Gentofte, Herlev, Denmark; Center for Advanced Heart Disease, Brigham and Women’s Hospital and Harvard Medical School, Boston, MA, USA; Mount Sinai Fuster Heart Hospital, Icahn School of Medicine at Mount Sinai, NewYork, NY, USA; Department of Vascular Medicine and Endocrinology, University Medical Centre Utrecht, Utrecht 3508 GA, The Netherlands; Department of Vascular Medicine and Endocrinology, University Medical Centre Utrecht, Utrecht 3508 GA, The Netherlands; Department of Cardiology, Thorax Center, Cardiovascular Institute, Erasmus MC, Rotterdam, The Netherlands; Department of Public Health and Primary Care, British Heart Foundation Cardiovascular Epidemiology Unit, University of Cambridge, Papworth Road, Cambridge Biomedical Campus, Cambridge, UK; Victor Phillip Dahdaleh Heart and Lung Research Institute, University of Cambridge, Cambridge, UK; National Institute for Health and Care Research Blood and Transplant Research Unit in Donor Health and Behaviour, University of Cambridge, Cambridge, UK; Health Data Research UK Cambridge, Wellcome Genome Campus and University of Cambridge, Cambridge, UK; Health Data Science Research Centre, Human Technopole, Milan, Italy; Department of Vascular Medicine and Endocrinology, University Medical Centre Utrecht, Utrecht 3508 GA, The Netherlands

**Keywords:** Heart failure, Secondary prevention, Established ASCVD, Risk prediction, Risk scores

## Abstract

**Background and Aims:**

Patients with established atherosclerotic cardiovascular disease (ASCVD) are at high risk of developing heart failure (HF). However, incident HF is not part of the risk assessment of current guideline-recommended models. The aim of this study was to develop and externally validate the SMART2-HF model for prediction of incident HF in patients with ASCVD.

**Methods:**

SMART2-HF was developed in 7698 individuals with established ASCVD (coronary, cerebrovascular, or peripheral artery disease, or abdominal aortic aneurysm) but without prior HF from the UCC-SMART cohort. Cox proportional hazards models including sex-predictor interactions and with age as the time scale were derived to estimate the 10-year and lifetime risk of incident HF (hospitalization for HF or HF-related death), accounting for competing non-HF mortality. Predictors, limited to routinely available clinical characteristics, were aligned with the SMART2 risk model for recurrent cardiovascular (CV) risk in the same population. External validation was performed in 240 741 patients with ASCVD from six data sources: the Clinical Practice Research Datalink, the HUNT3 study, the SWEDEHEART Registry, the ASCVD-Particles cohort, the Estonian Biobank and the international REACH Registry.

**Results:**

During a median follow-up of 11.2 years (interquartile range 6.1–16.4 years), 1031 incident HF events (13%) occurred in the UCC-SMART cohort. In the external validation data sources, a total of 24 885 incident HF events (10%) occurred. The pooled *C*-statistic was .696 (95% confidence interval .674–.717), with consistent performance in subgroups by sex and type of ASCVD. Predicted risks matched observed incidence in external validation.

**Conclusions:**

The SMART2-HF model enables the prediction of incident HF in patients with ASCVD. Aligned with the guideline-recommended SMART2 model for recurrent CV risk, SMART2-HF can be used as a complementary tool in this population.

## Structured Graphical Abstract

**Structured Graphical Abstract:**
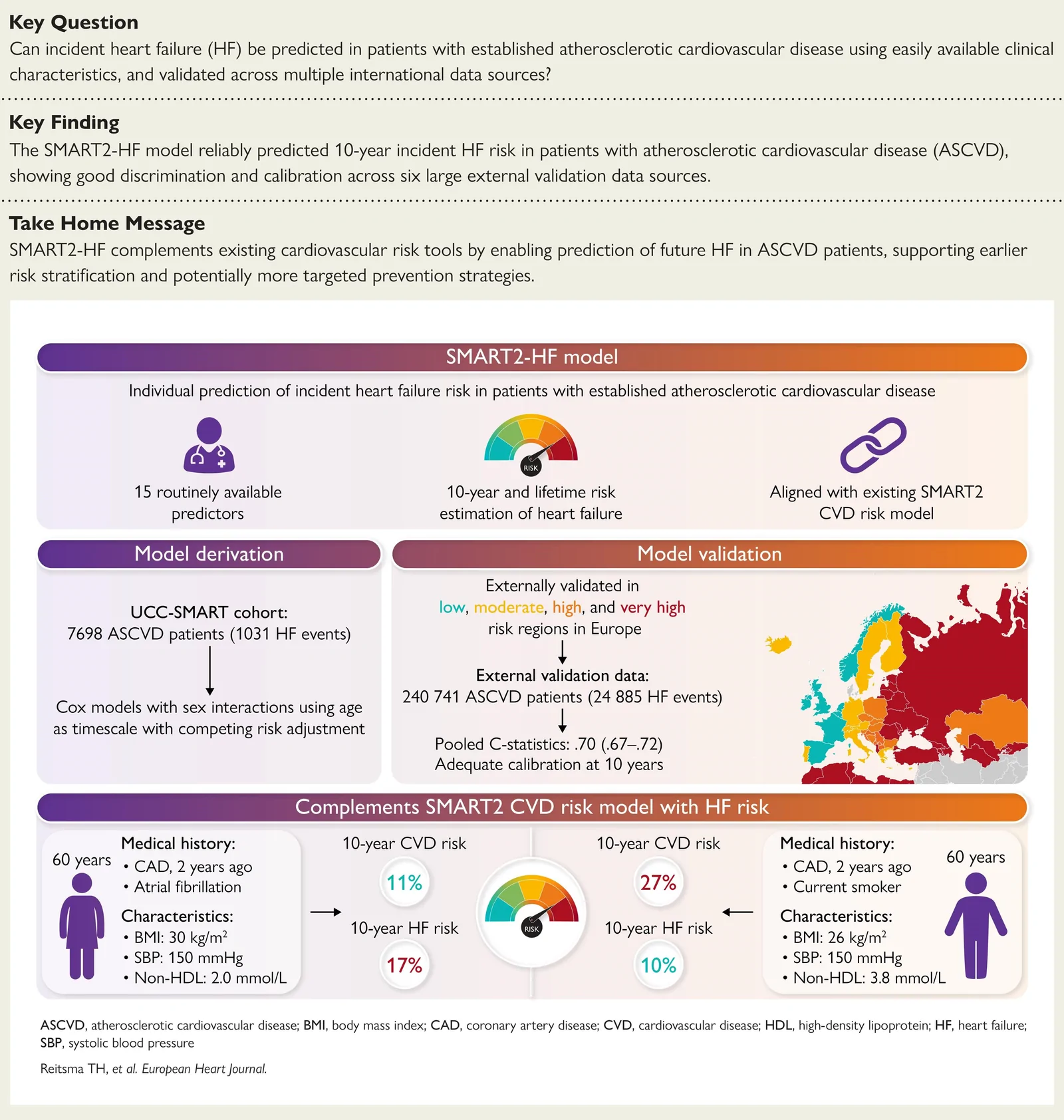
Graphical abstract of the derivation, validation and utility of the SMART2-HF model. ASCVD, atherosclerotic cardiovascular disease; BMI, body mass index; CAD, coronary artery disease; CVD, cardiovascular disease; HDL, high-density lipoprotein; HF, heart failure; SBP, systolic blood pressure; UCC-SMART, Utrecht Cardiovascular Cohort-Secondary Manifestations of ARTerial disease.

**See the editorial comment for this article ‘Risk scores for heart failure: powerful for populations, problematic for the individual patient’, by Christian Torp-Pedersen**  ***et al.*****, https://doi.org/10.1093/eurheartj/ehag183.**

## Introduction

Heart failure (HF) affects over 60 million people globally.^1^ Both incidence and prevalence are increasing in the context of the ageing population, rising rates of cardiovascular (CV) risk factors and improved survival for patients with atherosclerotic cardiovascular disease (ASCVD).^2–4^ Patients with established ASCVD have a higher risk of developing incident HF than the general population.^5–7^ Similar to the risk of recurrent CV events, the risk of incident HF is highly variable between individuals.^5^ The 2021 European Society of Cardiology (ESC) guidelines for CV disease prevention recommend using CV risk prediction models in the ASCVD population to decide upon (intensification of) preventive treatment.^8^ SMART and SMART-REACH models are recommended models for predicting 10-year and lifetime risks of recurrent CV events in patients with ASCVD and are validated in the European risk regions.^8–10^ The SMART model was developed using the Utrecht Cardiovascular Cohort—Secondary Manifestations of ARTerial disease (UCC-SMART) and externally validated in several trial and routine care populations.^10^ The model uses routinely available predictors to estimate the risk of non-fatal myocardial infarction, stroke and CV death. The successor of this model is the SMART2 model, which included several improvements including competing risk adjustment and regional recalibration to account for geographical differences in disease incidence.^11^

However, the composite endpoint of these scores is restricted to CV mortality, non-fatal myocardial infarction, and non-fatal stroke. Importantly, by not including incident HF, these risk scores underestimate total CV disease burden in this population. Furthermore, while HF risk correlates with CV risk, individuals may have substantially elevated HF risk despite low predicted risk of recurrent CV events.^12^ This discordance might be primarily attributable to HF-specific risk factors such as hypertension, obesity and atrial fibrillation that are not fully captured in the existing CV risk prediction models.^12,13^ Therefore, a complementary model, validated in the European risk regions, alongside existing risk scores is needed for the estimation of incident HF in individuals with ASCVD.^5^ Improving prediction of incident HF in patients with ASCVD could assist in early identification of patients at high risk of developing HF, thereby facilitating timely initiation of prevention strategies.

Therefore, the objective of the current study was to develop and externally validate the SMART2-HF model for individual prediction of incident HF risk in patients with established ASCVD without a previous HF diagnosis.

## Methods

### Study populations

The target population of the SMART2-HF risk model, in line with the SMART2 risk model for prediction of recurrent CV events, consists of individuals with stable, established ASCVD, but in contrast to SMART2 patients with a prior diagnosis of HF were excluded. The SMART2-HF risk model was developed using patients with established ASCVD from the UCC-SMART, an ongoing single-centre prospective cohort in University Medical Center Utrecht, The Netherlands.^14^ From 1996 onward, UCC-SMART includes patients referred to the University Medical Center Utrecht with or at high risk of ASCVD. At baseline, a standardized screening protocol was used to obtain medical history, perform physical examination, and conduct laboratory measurements.^14^ For the current study, UCC-SMART was linked to the national hospitalization registry and national causes of death registry from Central Bureau of Statistics in the Netherlands. These registries continuously collect causes of all hospitalizations and all registered cause of death reports in the Netherlands. Linkage with these registries was available until 31st of December 2023. Patients aged between 40 and 90 years who had a history of ASCVD and no history of hospitalization with HF were included. History of ASCVD was defined as coronary artery disease (CAD), cerebrovascular disease (CeVD), peripheral artery disease (PAD), and/or abdominal aortic aneurysm (AAA) from baseline questionnaires, enriched with previous hospitalization data (see Supplementary data online, *Table S1*). Individuals who could not be uniquely linked to the registries of Statistics Netherlands were excluded (see Supplementary data online, *Figure S1* for the flowchart of patient selection).

The model was externally validated in patients with ASCVD and without a history of HF in the SWEDEHEART (Swedish Web system for Enhancement and Development of Evidence based care in Heart disease Evaluated According to Recommended Therapies) registry in Sweden,^15^ the Norwegian HUNT3 study (Helse Undersøkelsen i Trøndelag 3),^16^ Clinical Practice Research Datalink (CPRD) in the United Kingdom,^17^ the Estonian Biobank,^18,19^ the ASCVD-Particles cohort in Poland,^20^ and the Reduction of Atherothrombosis for Continued Health (REACH) registry.^21–24^ Individuals up to age of 80 years were included in the external validation analyses in order to validate 10-year predicted risks. Detailed description of the external validation data sources has been published previously and is summarized in the Supplementary Methods, including patient flowcharts for SWEDEHEART and CPRD. The ASCVD and HF definitions used at baseline in all data sources are described in Supplementary data online, *Table S1*. Outcomes were assessed through annual questionnaires (REACH registry) or through linkage with hospital records or national disease/mortality registries (CPRD, SWEDEHEART, HUNT3, Estonian Biobank, ASCVD-Particles).

### Outcomes and predictors

To enable synergistic use next to the SMART2 model, which already captures recurrent CV events (including CV mortality), the primary outcome of the SMART2-HF model was incident HF (both with reduced and preserved ejection fraction). Incident HF was defined as the composite outcome of first hospitalization with HF or HF death. Hospitalizations with HF were defined as a hospitalization with HF registered as diagnosis in any position (i.e. primary or subsequent diagnostic positions). As such, the outcome reflects clinically manifest HF events identified at hospital level, including HF recorded as comorbid diagnosis. HF death was defined as mortality with HF recorded as primary or secondary cause of death. Non-HF death was considered as the competing outcome event and defined as all mortality without HF as primary or secondary cause. Detailed definitions of outcomes, including the specific International Classification of Diseases 10th Revision (ICD-10) codes, are described in Supplementary data online, *Table S2*.

In order to allow for parallel estimation of risk of recurrent CV events (using the SMART2 risk model^11^) and risk of incident HF, all predictors used by the SMART2 risk model were also selected for SMART2-HF: age, sex, current smoking, diabetes mellitus, systolic blood pressure, non-high density lipoprotein (HDL) cholesterol, presence of CAD, presence of CeVD, presence of PAD, presence of AAA, estimated glomerular filtration rate (based on creatinine using the 2009 Chronic Kidney Disease Epidemiology Collaboration equation^25^), C-reactive protein (CRP), and years since first ASCVD diagnosis (CAD, CeVD, PAD, or AAA) (see Supplementary data online, *Table S3*). Two additional predictors were added based on the clinical relevance in the development of incident HF as judged by an expert panel and availability in clinical practice: history of atrial fibrillation and body mass index (BMI). Preventive treatment options were not considered as predictors because the predictive value of treatment options is strongly influenced by clinician judgement and changing treatment patterns.^26^

### Model development

Cause-specific Cox proportional hazards functions were derived for incident HF and non-HF death, including interactions between all predictors and sex to account for sex-specific differences in relative effects of predictors. Age was used as the underlying timescale, taking age at entry into the cohort as starting point (left truncation) up to age of censoring or event (right censoring). Using age as timescale allows for estimation of age-specific probabilities for incident HF over the entire age-range of the cohort (40–90 years).^27^ To account for effects of age-of-entry into the cohort, age at inclusion was included as a predictor in the model.^28^

Continuous predictors were truncated at 1st and 99th sex-specific percentiles to limit effects of outliers. Non-linear transformations for continuous predictors were applied if they improved model fit based on Akaike’s Information Criterion (details in Supplementary Methods). Interactions with baseline age were added in case of violations of proportional hazards. Ridge regression was applied to shrink all coefficients and reduce overfitting of the model (Supplementary Methods). Baseline survival probabilities for both outcomes were derived for 1-year age intervals and smoothed using non-linear functions (see Supplementary Methods).

In the derivation data, missing data were imputed using the multivariate imputation by chained equations (R-package *mice*) with five imputations and five iterations. Both outcomes (incident HF and non-HF death) were included using Nelson–Aalan estimators and event indicators. Further information on missing data is reported in Supplementary data online, *Table S4* and in the Supplementary Methods.

### Individual and lifetime risk prediction

Individual and lifetime (e.g. risk until 90 years) risk of incident HF and non-HF death was estimated using previously applied lifetable methods.^9,27,29^ In a lifetable, all remaining 1-year survival probabilities until age 90 for both outcomes are estimated by combining the coefficients with the smoothed baseline survival probabilities. Combining the survival probabilities for both outcomes accounts for competing risks. Interval survival probabilities were multiplied to calculate the cumulative probability of reaching a specific age free of any event. HF-free life expectancy is defined as the age at which this predicted cumulative event-free survival probability drops below 50%. As a result, the SMART2-HF model can estimate any year (including 10-year) and lifetime risk of incident HF as well as HF-free life expectancy. Detailed descriptions of statistical methods are provided in the Supplementary Methods.

### Regional recalibration

The SMART2-HF risk model was recalibrated to CV risk regions within Europe, which were previously grouped based on age- and sex-standardized CV disease mortality rates for the SMART2 model.^11^ The four risk regions are low-risk, moderate-risk, high-risk. and very-high risk (see Supplementary data online, *Figure S2*). The model was recalibrated to these risk regions within Europe by recalibrating the baseline hazard of the SMART2-HF risk model using the expected-observed (EO) ratio (a single multiplicative constant per region) as recalibration factor from the data source deemed most representative of the region (details in Supplementary Methods). Recalibration was performed using EO ratios from 10-year predicted risks, where available. For the low-risk region, CPRD (*n* = 110 309) and for the moderate-risk region, SWEDEHEART (*n* = 75 353) were population-based data sources deemed representative for their risk regions. For the high-risk region, the ASCVD-Particles cohort (*n* = 1456) was used for recalibration, as it represented the most suitable dataset available to us with robust long-term follow-up. For the very high risk region, no data source with reliably adjudicated, long-term follow-up was available that could support recalibration. Instead, the recalibration factors from the high-risk region were used for the very high-risk region as well. The REACH registry could not be used for this purpose because incident HF was not continuously recorded; only visit-level yes/no information was available.^21–24^ Consequently, REACH was used solely to assess model discrimination rather than calibration or recalibration.

### External validation

Model performance was assessed by discrimination, using Harrell's *C*-statistic, and calibration, by using calibration plots comparing predicted risks vs observed incidence, calibration slopes and intercepts, and EO ratios. *C*-statistics and calibration measures were adjusted for competing risks by using competing-risk adjusted cumulative incidences (see Supplementary Methods). Where possible, model performance was assessed at 10 years of follow-up. For data sources with less than 10 years of follow-up (ASCVD-Particles, REACH, Estonian Biobank), model performance was assessed at the last full year in which at least 75th percentile was still in follow-up in the respective data source (9 years for ASCVD-Particles, 5 years for Estonian Biobank, 2 or 3 years for the REACH, depending on the region). For model discrimination, a pooled *C*-statistic was calculated across all external validation data sources using inverse-variance weighting.^30^  *C*-statistics of SMART2-HF were compared with *C*-statistics of the previously developed PREVENT-HF equations for incident HF in the general population,^31^ as no guideline-recommended equations are yet available for the ASCVD population. Model performance was assessed separately across the pre-defined sub-groups of sex and across the different subtypes of ASCVD (i.e. CAD, CeVD, PAD, AAA), and patients with two or more ASCVD diagnoses at baseline, referred to as polyvascular disease. To evaluate the clinical benefit of SMART2-HF, decision curve analyses were performed in data sources with long-term follow-up (CPRD, HUNT3, SWEDEHEART, and ASCVD-Particles). For this purpose, the net benefit of individualized treatment guided by SMART2-HF was compared with strategies of treating all patients or treating none across a range of clinically relevant treatment thresholds.

### Sensitivity analyses

As a sensitivity analysis, model development was repeated in UCC-SMART separately for men and women to assess whether sex-specific models improved performance. Second, to assess whether limited sample size in the derivation cohort affected external performance, an identically developed model in SWEDEHEART was derived, which has a much larger sample size than UCC-SMART yet consists of post-myocardial infarction patients only. *C*-statistics of both alternative models were compared with the SMART2-HF model in two external validation data sources (HUNT3 and ASCVD-Particles). Third, three sensitivity analyses were conducted during model derivation. To assess the impact of potentially missed prevalent HF, model derivation was repeated after excluding incident HF events occurring within the first 6 months. To evaluate the contribution of HF-related death, HF death without a preceding HF hospitalization was not counted as an incident HF event. To assess the impact of myocardial infarction preceding HF onset, individuals experiencing a myocardial infarction during follow-up were censored at the time of infarction. Coefficients from the primary model were compared with those obtained from each of these sensitivity analyses.

### Clinical utility

To illustrate the added clinical relevance of the SMART2-HF model alongside the existing SMART2 model,^11^ 10-year predicted risks for incident HF and recurrent CV events were calculated for individuals in HUNT3 using the SMART2-HF and SMART2 risk models, respectively. Individuals were stratified into sex-specific quarters of predicted SMART2 risk and within each quarter the distribution of predicted SMART2-HF risk was examined. A potential application of the SMART2-HF model is the estimation of individualized benefit from preventive treatment, which is further described in the Supplementary Methods.^27,29,32^

All analyses were performed with R-statistical programming (version 4.3.2, R Foundation for Statistical Computing, Vienna, Austria). Reporting was performed in accordance with TRIPOD statement^33^ (checklist in Supplements).

## Results

### Baseline characteristics

For model derivation, 2065 women and 5633 men with established ASCVD were included from the UCC-SMART cohort. Median age at baseline was 61 [interquartile range (IQR) 53–69] years for women and 62 (IQR 55–68) years for men. Four types of ASCVD were represented in the derivation population (CAD 65%, CeVD 30%, PAD 16%, and AAA 7%) and 1201 (16%) patients had polyvascular disease. Incident HF occurred in 1031 patients (13%) during a median follow-up of 10.6 years (IQR 5.6–15.9 years). The majority of incident HF events were captured by hospitalizations (96%) and 15% of patients experienced a preceding CV event prior to onset of HF, most commonly myocardial infarction (see Supplementary data online, *Table S5*). Non-HF mortality occurred in 2110 patients (27%) during a median follow-up of 11.2 years (IQR 6.1–16.4 years). Detailed patient characteristics stratified by sex are presented in *Table 1*.

**Table 1:** Sex-specific baseline characteristics of the UCC-SMART study population

	Women *n* = 2065	Men *n* = 5633
Age (years)	61 [53–69]	62 [55–68]
Current smokers	625 (30)	1443 (26)
Physical examination		
Systolic blood pressure (mmHg)	136 [123–152]	136 [125–149]
Diastolic blood pressure (mmHg)	78 [71–87]	80 [74–88]
Body mass index (kg/m^2^)	26 [24–29]	27 [25–29]
Medical history		
Coronary artery disease	996 (48)	3989 (71)
Cerebrovascular disease	861 (42)	1464 (26)
Peripheral artery disease	410 (20)	800 (14)
Abdominal aortic aneurysm	91 (4)	449 (8)
Polyvascular disease^a^	264 (13)	937 (17)
Diabetes mellitus	341 (16)	969 (17)
Atrial fibrillation	88 (4)	477 (8)
Years since first ASCVD diagnosis	.5 [.0–3.0]	1.0 [.2–5.5]
Laboratory measurements		
Total cholesterol (mmol/L)	4.8 [4.1–5.8]	4.4 [3.7–5.3]
LDL cholesterol (mmol/L)	2.7 [2.0–3.5]	2.5 [1.9–3.2]
HDL cholesterol (mmol/L)	1.4 [1.2–1.7]	1.1 [.9–1.3]
Non-HDL cholesterol (mmol/L)	3.3 [2.6–4.3]	3.2 [2.5–4.1]
Triglycerides (mmol/L)	1.3 [1.0–1.8]	1.4 [1.0–2.0]
CRP (mg/L)	2.1 [1.0–4.4]	1.8 [.9–3.7]
Estimated GFR (mL/min/1.73 m^2^)	76 [64–88]	81 [69–92]
Use of medication		
Antihypertensives	1505 (73)	4417 (78)
Lipid lowering therapy	1361 (66)	4323 (77)
Antiplatelet therapy or anticoagulants	1621 (78)	4944 (88)
Outcomes		
Incidence of HF	234 (11)	797 (14)
Event rate per 1000 person-years	10	13
Follow-up time (years)	10.9 [6.0–16.3]	10.4 [5.5–15.7]
Incidence of non-HF death	531 (26)	1579 (28)
Event rate per 1000 person-years	22	25
Follow-up time (years)	11.5 [6.3–16.6]	11.1 [6.1–16.3]

Data are presented as *n* (%) or median [25th–75th percentile].

ASCVD, atherosclerotic cardiovascular disease; LDL, low-density lipoprotein; HDL, high-density lipoprotein; CRP, C-reactive protein; GFR, glomerular filtration rate (calculated with the 2009 Chronic Kidney Disease Epidemiology Collaboration formula); HF, heart failure.

^a^Polyvascular disease was defined as having two or more ASCVD diagnoses at baseline.

### Model derivation

The penalized hazard ratios for incident HF and non-HF death of the SMART2-HF model are presented in Supplementary data online, *Table S6*. No violations of the proportional hazards assumption were observed and no interactions with age were required. All parameters needed for individual risk estimation are shown in the Supplementary Material; age-specific baseline survival probabilities are presented in Supplementary data online, *Table S7* and Supplementary data online, *Figure S3*, and equations for linear predictors are provided in Supplementary data online, *Table S8*. Internal validation *C*-statistics were .748 [95% confidence interval (CI) .726–.769] for incident HF and .751 (95% CI .729–.774) for non-HF mortality. Internal calibration at 10 years showed alignment between predicted risks and observed incidence, consistent across sexes (see Supplementary data online, *Figure S4*).

### Model validation

**Figure 1:**
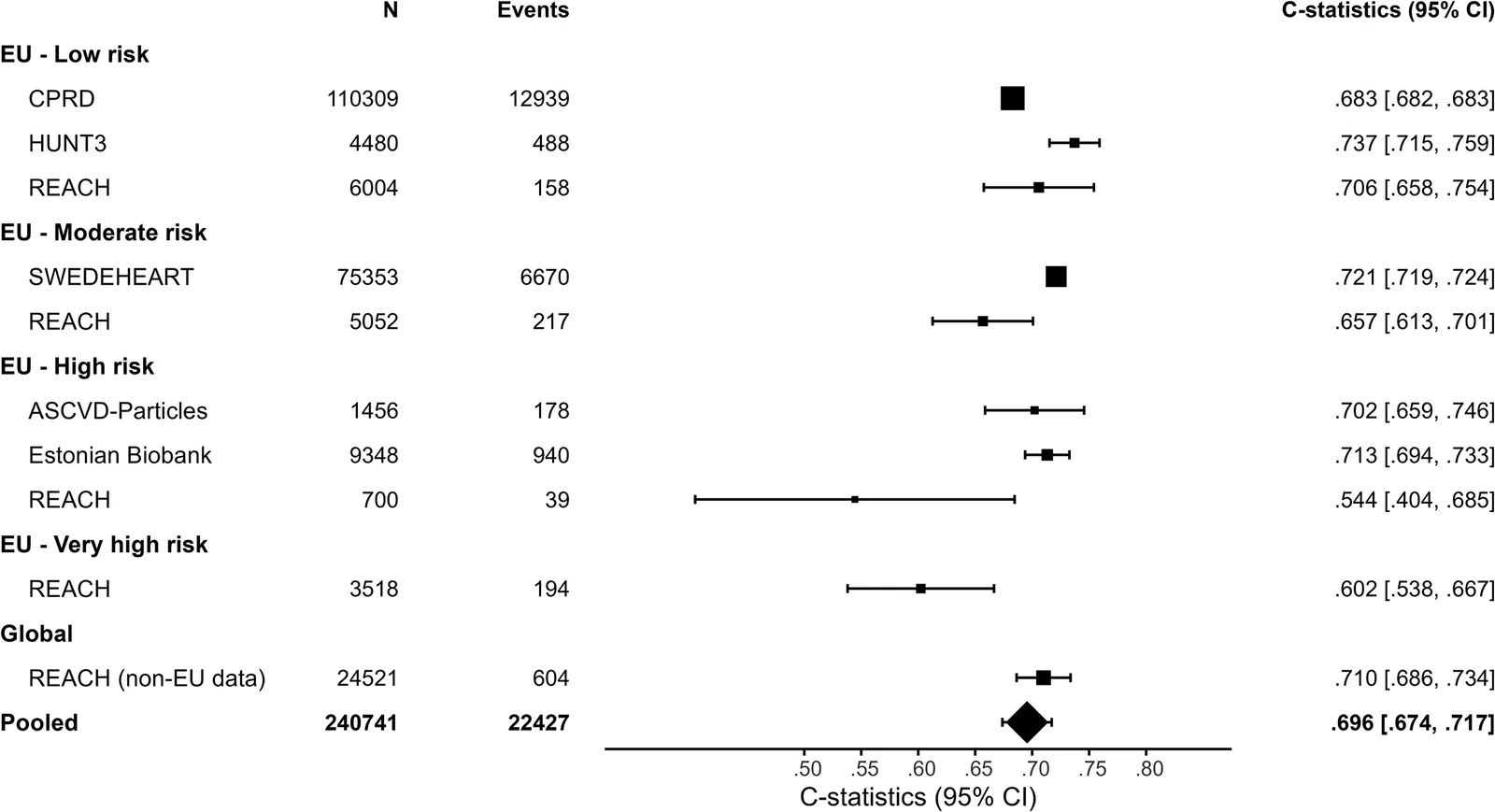
Discrimination for incident HF as measured by *C*-statistic in external validation. Discrimination in all external validation data is based on Harrell’s *C*-statistic. Discrimination was assessed at 10 years (CPRD, HUNT3, SWEDEHEART) or at the 75th percentile of follow-up duration for ASCVD-Particles (9 years), Estonian Biobank (5 years) and REACH (2 or 3 years, depending on the region). EU, Europe; CI, confidence interval

External validation of the SMART2-HF model involved 240 741 individuals with established ASCVD from six data sources, representing different European risk regions. In total, 24 885 (10.3%) incident HF events occurred. Median follow-up ranged from 1.8 years (IQR 1.6–1.9) in REACH to 12.0 years (IQR 9.8–12.6) in HUNT3. Detailed patient characteristics of the participants included in the external validation data sources are presented in *Table 2*. Pooled *C*-statistic across all external validation data sources was .696 (95% CI .674–.717) and consistent across risk regions (*Figure 1*). The *C*-statistics of the SMART2-HF model were consistent for men and women and across different types of ASCVD (see Supplementary data online, *Figures S5* and *S6*). *C*-statistics of SMART2-HF were consistently higher than the PREVENT-HF equations, showing a statistically significant difference in almost all external validation data sources (see Supplementary data online, *Table S9*).

**Table 2:** Baseline characteristics of external validation data sources

	CPRD *n* = 110 309	HUNT3 *n* = 4480	SWEDEHEART *n* = 75 353	ASCVD-Particles *n* = 1456	Estonian Biobank *n* = 9348	REACH *n* = 39 795
Country	UK	Norway	Sweden	Poland	Estonia	Multiple
Risk region	EU Low risk	EU Low risk	EU Moderate risk	EU High risk	EU High risk	EU Low–very high risk and non-EU
Age (years)	69 [62–75]	66 [60–73]	64 [57–70]	66 [59–72]	65 [58–72]	67 [60–73]
Men	68 713 (62)	2602 (58)	55 840 (74)	735 (50)	3714 (40)	27 633 (69)
Current smokers	29 505 (27)	803 (18)	8576 (11)	88 (6)	1262 (14)	6463 (16)
Physical examination						
Systolic blood pressure (mmHg)	136 [125–145]	134 [122–147]	130 [120–140]	135 [123–150]	134 [122–145]	135 [124–150]
Body mass index (kg/m^2^)	28 [25–31]	28 [25–31]	27 [25–30]	28 [25–32]	28 [25–31]	27 [24–30]
Medical history						
Coronary artery disease	76 811 (70)	2813 (63)	75 353 (100)	1360 (93)	7677 (82)	27 649 (70)
Cerebrovascular disease	30 622 (28)	1263 (28)	2691 (4)	186 (13)	2341 (25)	13 506 (34)
Peripheral artery disease	16 164 (15)	1002 (22)	2359 (3)	223 (15)	1994 (21)	5796 (15)
Abdominal aortic aneurysm	2692 (2)	^a^	824 (1)	8 (1)	140 (2)	1185 (3)
Polyvascular disease	14 536 (13)	540 (12)	5422 (7)	207 (14)	422 (5)	909 (2)
Diabetes mellitus	20 983 (19)	591 (13)	15 882 (21)	423 (29)	1582 (17)	14 217 (36)
Atrial fibrillation	^a^	424 (10)	6491 (9)	168 (12)	1444 (15)	2934 (7)
Years since first ASCVD diagnosis	7.0 [3.3–12.1]	7.3 [3.3–13.3]	.2 [.1–.2]	^a^	7.2 [3.7–12.0]	1.0 [.5–1.0]
Laboratory measurements						
Non-HDL cholesterol (mmol/L)	3.1 [2.5–3.7]	3.7 [3.1–4.6]	2.5 [2.0–3.1]	3.3 [2.6–4.0]	^a^	3.6 [2.9–4.4]
CRP (mg/L)	1.8 [1.8–2.1]	1.5 [.8–3.3]	4.0 [2.0–6.0]	^a^	2.0 [1.0–5.0]	^a^
Estimated GFR (mL/min/1.73 m^2^)	71 [60–84]	89 [76–97]	98 [90–104]	87 [74–97]	87 [74–98]	73 [58–88]
Incidence of HF	13 707 (12)	628 (14)	7291 (10)	201 (14)	1562 (17)	1496 (4)
Follow-up time (years)	7.0 [3.7–9.5]	12.0 [9.8–12.6]	5.1 [2.2–8.6]	7.8 [6.5–9.3]	5.1 [4.6–5.8]	1.8 [1.6–1.9]
Event rate per 1000 person-years	18.9	13.3	17.1	18.6	26.7	19.4

Data are presented as *n* (%) or median [25th–75th percentile].

UK, United Kingdom; EU, Europe; ASCVD, atherosclerotic cardiovascular disease; HDL, high-density lipoprotein; CRP, C-reactive protein; GFR, glomerular filtration rate (calculated with the 2009 Chronic Kidney Disease Epidemiology Collaboration formula); HF, heart failure.

^a^Data for this variable are missing completely in this data source.

**Figure 2:**
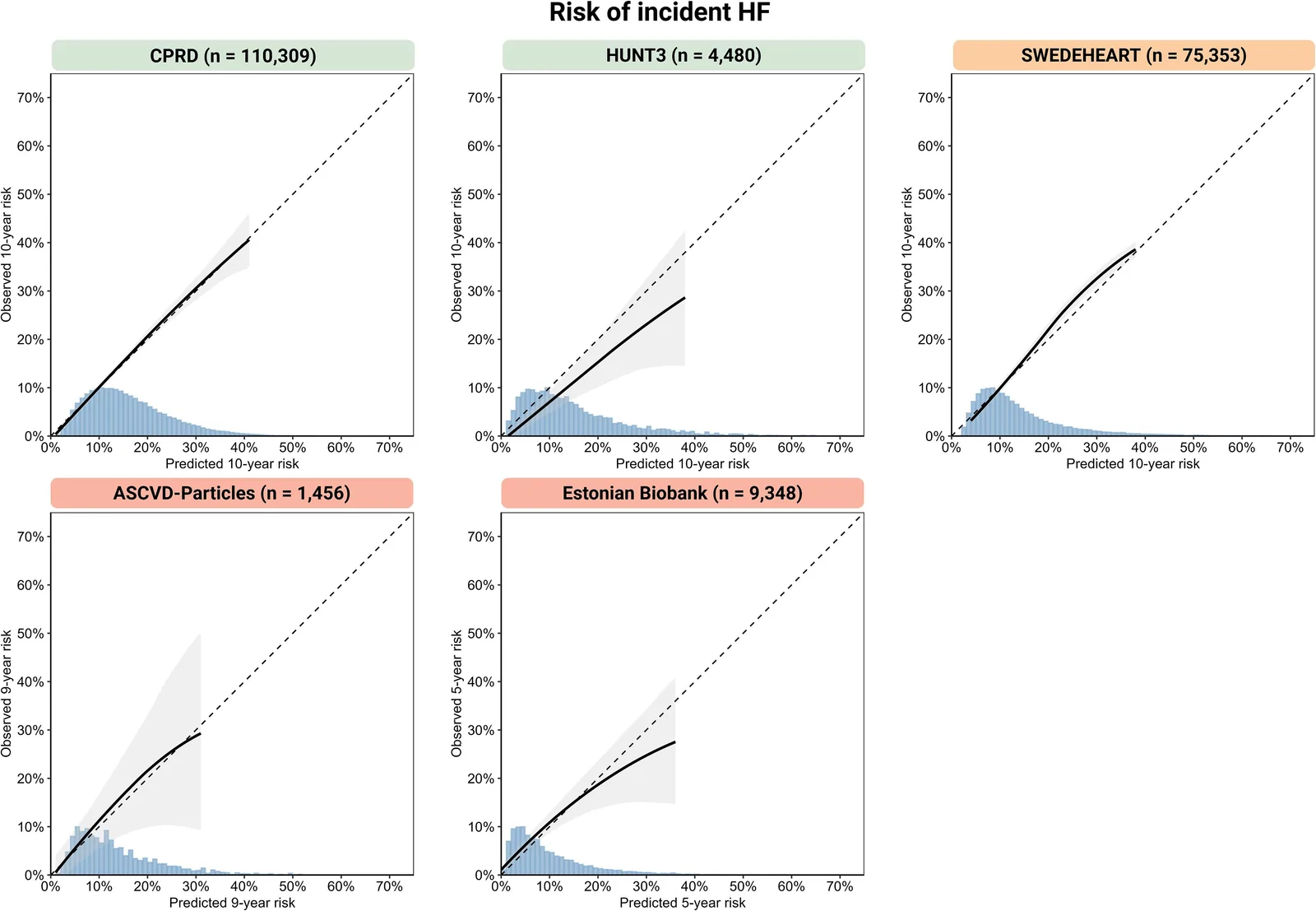
Calibration for incident HF in external validation. Calibration of the SMART2-HF model in external validation data sources representing European low- (CPRD and HUNT3), moderate- (SWEDEHEART), and high-risk (ASCVD-Particles and Estonian Biobank) regions. Smoothed calibration plots of predicted risks vs observed incidence and a histogram of the predicted risks (in blue) is shown. Calibration is shown at 10 years where possible, or at the 75th percentile of follow-up duration for ASCVD-Particles (9-year risks) and Estonian Biobank (5-year risks). HF, heart failure

Regional recalibration was performed for European low-, moderate-, and high-risk regions and recalibration factors can be found in Supplementary data online, *Table S10*. After recalibration, calibration was adequate across these risk regions (*Figure 2*). Overall, calibration was comparable in men and women (see Supplementary data online, *Figures S7* and *S8*). EO ratios, calibration intercepts, and calibration slopes in all external validation data sources are presented in Supplementary data online, *Table S11*. Calibration for non-HF death was adequate across in most external validation data sources, only in the Estonian Biobank an overestimation of predicted risks was observed (see Supplementary data online, *Figure S9*). Decision curve analyses showed that individualized treatment based on SMART2-HF resulted in higher clinical benefit than the treat-all or treat-none strategies in four external validation data sources for treatment thresholds from 15% to 30% (see Supplementary data online, *Figure S10*).

### Sensitivity analyses

Repeating model development for men and women separately in UCC-SMART yielded lower discrimination than the SMART2-HF model in external validation (see Supplementary data online, *Figure S11*). Using SWEDEHEART instead of UCC-SMART for model development did not improve external performance. Discrimination was similar for women and higher for men with the UCC-SMART model, supporting adequacy of the UCC-SMART sample size (see Supplementary data online, *Figure S11*). Repeating model development after exclusion of incident HF events occurring in the first 6 months resulted in 2.2% reduction in incident HF events and showed consistent model coefficients (see Supplementary data online, *Table S12*). Similarly, sensitivity analyses excluding HF deaths from the outcome and censoring patients who experienced an intermittent myocardial infarction prior to their incident HF event resulted in 3.9% and 10% reductions in incident HF events, respectively, with consistent model coefficients (see Supplementary data online, *Table S12*).

### Clinical utility

**Figure 3:**
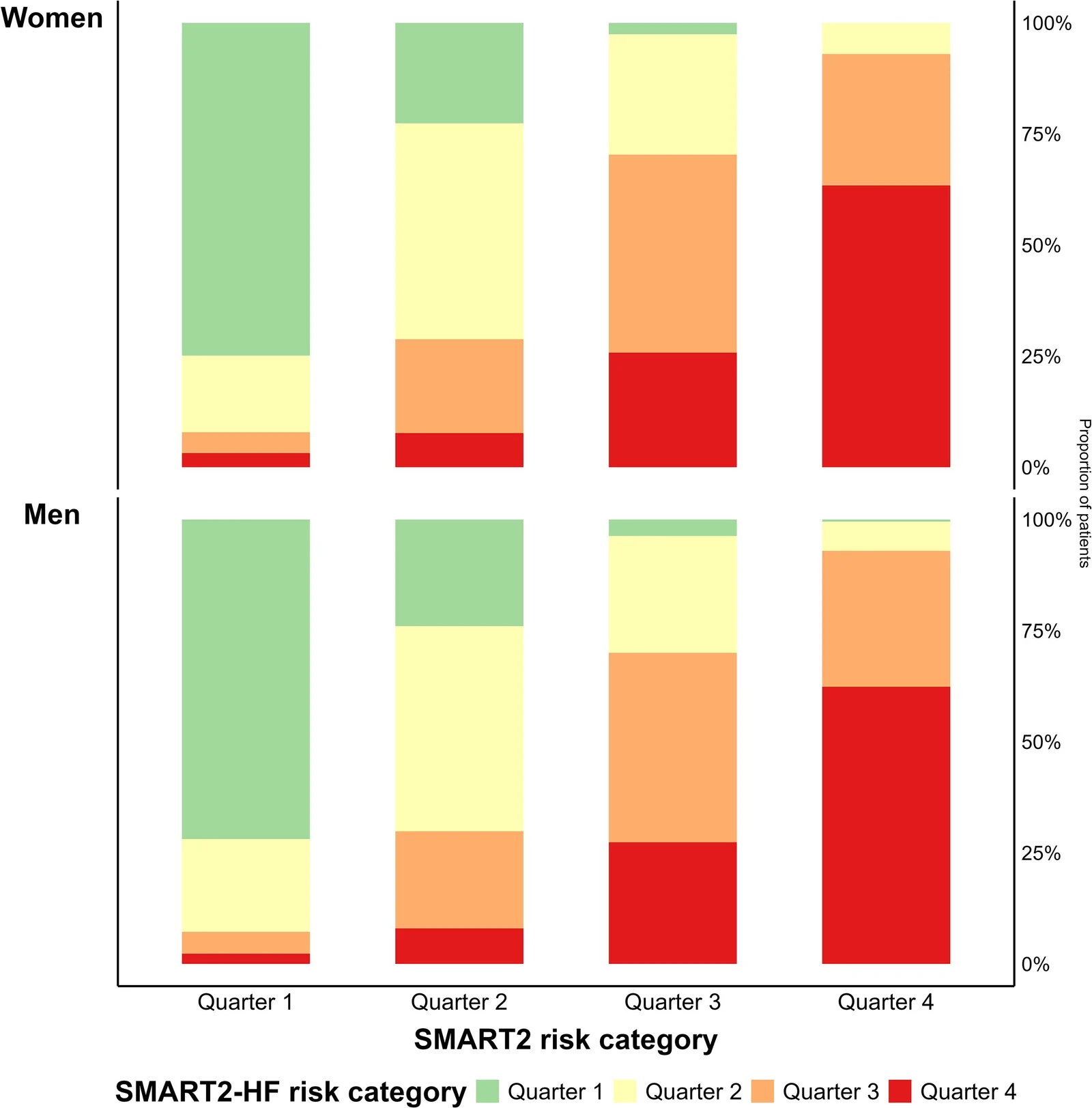
Distribution of SMART2-HF predicted incident HF risks, stratified by quarters of SMART2 predicted recurrent CV risks. The figure shows the distribution of 10-year incident HF risks (estimated with SMART2-HF) within quarters of CV risks (estimated with SMART2) in the validation population of HUNT3 (low risk region). For both models, patients were divided into four risk categories based on the sex-specific 25th, 50th, and 75th percentiles of the respective risk distributions. For SMART2-HF, the corresponding risk thresholds defining the sex-specific quarters were 5%, 10%, and 17% for women and 9%, 14% and 22% for men. For SMART2, the sex-specific quarter thresholds were 14%, 20%, and 27% for women and 18%, 25%, and 34% for men. Histograms of the continuous risk distributions for both models in HUNT3 are shown in Supplementary data online, *Figure S12*. Colours are used for illustrative purposes only

Overall, SMART2 predicted 10-year CV risks were higher than SMART2-HF predicted risks in both women and men (see Supplementary data online, *Figure S12*). Individuals at high risk of recurrent CV events had generally higher HF risks (*Figure 3*). However, among both women and men in the lowest SMART2 risk category, ∼ 25% were classified in higher SMART2-HF risk categories, indicating relatively higher HF risk despite low predicted CV risk.

In Supplementary data online, *Figure S13*, two clinical examples for the use of the SMART2-HF model are presented, including an estimation of individualized treatment benefit. An example of the online calculator for clinical use can be accessed (https://tessa-r.shinyapps.io/smart2-hfapp/).

## Discussion

The current paper describes the development and external validation of the SMART2-HF model for individual prediction of incident HF in patients with established ASCVD. The SMART2-HF model allows for estimation of both 10-year and lifetime risk of incident HF, as well as estimation of HF-free life expectancy. The model was externally validated across European and global risk regions and performed well in terms of discrimination and calibration (see *Structured Graphical Abstract*).

Risk prediction for guiding preventive treatment in ASCVD patients has been a key focus in recent CV disease guidelines.^8,34^ While various validated prediction models exist for estimation of the risk of recurrent CV events, prediction models for estimation of the risk of incident HF are scarce, especially in the ASCVD population. Risk estimation of HF in ASCVD patients can assist in early identification of patients at high risk of developing HF. In these patients, more intensive risk factor management could be considered, such as weight reduction, intensive blood pressure lowering, sodium–glucose cotransporter 2 inhibitors, and incretin therapy (e.g. glucagon-like peptide-1 receptor agonists).^35–39^ The SMART2-HF model could identify a high-risk population with the largest absolute benefit from preventive treatment and therefore optimize the deployment of (expensive) lifelong therapies. In addition, the SMART2-HF model could aid in deciding upon referral to specialty clinics for management for patients currently managed in primary care. Lastly, risk prediction based on the SMART2-HF model may facilitate shared decision-making and increase motivation for drug treatment and lifestyle changes, thereby optimizing adherence.^40^

The SMART2-HF model has features that offer several advantages compared with existing models. First, the SMART2-HF model is supported by derivation data that included different types of ASCVD and has a long follow-up period. In addition, SMART2-HF has been thoroughly externally validated across different data sources from multiple countries. This offers greater applicability of the derived model. The populations used for derivation and validation also included patients with polyvascular disease (i.e. patients with ASCVD at multiple locations) making application to this high-risk population possible.

Second, guidelines recommend the use of prediction models like SMART2 for more tailored CV risk prediction in the ASCVD population.^8,11^ Yet, these tools currently do not capture the full burden of CV disease, as they do not include incident HF. An important strength of the SMART2-HF model is the use of the same predictors as in the guideline-recommended SMART2 risk model for CV risk predictions. Only two additional predictors, BMI and atrial fibrillation, were added to improve HF-specific prediction. Aligning with the SMART2 risk model enables parallel estimation of HF and CV risk, with minimal additional effort. Importantly, predicting incident HF separately provides complementary insights as it identifies partly distinct high-risk individuals who may benefit from targeted prevention or monitoring strategies, illustrated by the differences between high HF risk and high CV risk categories in *Figure 3*. As a result, the SMART2-HF model complements the SMART2 risk model to give a more exhaustive view of the total CV risk of an individual. Using the holistic information about an individual’s HF risk and CV risk may help improve shared decision-making in clinical practice. In addition, by aligning with the SMART2 risk model, SMART2-HF can be easily implemented in software calculators, such as the ESC CVD risk calculation app and the CE marked medical device www.U-Prevent.com. Facilitating the parallel implementation on these platforms enables easy implementation and seamless integration into the clinical workflow.

Third, unlike previously developed risk models for predicting incident HF in individuals with established ASCVD, the SMART2-HF model accounts for the impact of competing risks.^5^ Because the risk predictions are intended for patients aged 40–80 years, not accounting for competing risk would lead to overestimation of risk and treatment effects, especially in high-risk populations such as individuals with established ASCVD.^41^ In addition, existing risk equations can only predict up to a maximum of 10-year risks. However, as age is the most important driver of both CV and HF risks, younger individuals typically have low 10-year risks but could have substantial benefits from preventive efforts on a lifetime perspective, which is incorporated in the SMART2-HF model.^27^

Fourth, model derivation with sex-predictor interactions allows SMART2-HF to capture sex-related variations in relative effects of predictors. The model with sex interactions for all predictors outperformed a completely sex-specific model, likely due to the more efficient use of power. For both sexes, SMART2-HF showed meaningful risk stratification within CV risk categories (estimated by the SMART2 model). This highlights the added value of complementary risk prediction of incident HF next to recurrent CV events.

The clinical value of HF risk prediction models lies in their ability to support meaningful clinical decisions through accurate and actionable absolute risk estimates. In external validation, SMART2-HF showed good calibration across all datasets, enabling reliable risk communication and shared decision making in clinical practice. In addition, SMART2-HF demonstrated improved discriminative performance compared with the PREVENT-HF equations and consistent performance across external validation data sources. Accordingly, SMART2-HF is best positioned as a tool to inform risk stratification and shared-decision making, rather than to serve as a stand-alone decision rule. The absolute value of the *C*-statistic should be interpreted with caution, as it depends not only on model quality but also on the underlying risk distribution.^42^ Decision curve analyses further demonstrated a clear net benefit of SMART2-HF across a clinically relevant range of decision thresholds, supporting its clinical usefulness.

Potential limitations of the SMART2-HF model need to be considered. First, the derivation of the SMART2-HF model was based on a cohort from a single Western European country with limited sample size, especially among women. Nevertheless, UCC-SMART was considered the most suitable dataset, as it includes patients with all major types of ASCVD, offers standardized measurements with minimal missing data, and provides long-term, complete follow-up. These strengths allowed reliable estimation of predictor–outcome associations, as reflected by adequate external discrimination, which was not improved in sensitivity analyses deriving the model in the substantially larger SWEDEHEART registry. Furthermore, recalibration was performed in more powerful representative data sources from three European risk regions, ensuring that the model is tailored to the local incidence across these European risk regions, in which adequate performance of the model was shown. Model performance in external validation confirmed robustness of the derived coefficients. As current validations for the very-high risk region in Europe and outside Europe were only performed in the REACH registry, with relatively short follow-up (2–3 years depending on region), future studies with longer follow-up in these regions are needed to further improve the geographic generalizability and regional calibration of the SMART2-HF model. In addition, validation data sources predominantly consisted of individuals of White European ancestry, which may limit the generalizability of SMART2-HF to other ethnic groups within Europe. Further validation in ethnically diverse populations could be addressed in future research.

Second, we used data on HF hospitalizations and HF-related mortality based on ICD-10 codes to define incident HF. Although HF hospitalizations accurately represent the acute care for HF,^43^ incident HF diagnosed in the outpatient or primary care setting is only indirectly captured if individuals are subsequently hospitalized for HF (primary diagnostic code) or other reasons (HF recorded in lower diagnostic position). This may result in some underestimation of the absolute HF risk in the population. However, because the majority of HF patients are ultimately identified through a hospital admission, this underestimation is likely to be relatively minor.^44,45^ In addition, by also including non-primary ICD-10 codes as incident HF, we were able to identify HF recorded as a comorbid condition during hospitalizations for other reasons, thereby capturing a substantial proportion of HF initially diagnosed in outpatient settings. As a result, SMART2-HF primarily predicts clinically manifest HF requiring hospital-level care. Another limitation of this study is that we could not distinguish between incident HF with preserved vs reduced ejection fraction, as such outcome data were not consistently available across the used data sources. Future extensions of SMART2-HF may enable phenotype-specific refinement of HF risk prediction and further enhance its clinical utility in this population.

Third, the SMART2-HF model was deliberately restricted to routinely available clinical predictors to ensure broad applicability and scalability. While this supports wide implementation across different clinical settings, the inclusion of additional HF-specific markers such as natriuretic peptides or echocardiographic parameters may further enhance predictive accuracy on top of SMART2-HF. This would allow for context-specific risk enrichment in clinical settings where such measures are routinely collected. In addition, SMART2-HF estimates risk based on baseline factors assumed to remain stable over time; however, as treatment or risk profiles may change in practice, re-estimation of predicted risk can be considered following major clinical changes.

In conclusion, this paper describes the SMART2-HF risk model predicting incident HF in patients with established ASCVD, externally validated in four European risk regions. SMART2-HF is aligned with the guideline-recommended SMART2 risk model for recurrent CV events and enables complementary use, allowing clinicians to assess HF and CV risk simultaneously and supporting more targeted prevention strategies in this high-risk population.

## Members of ESC Cardiovascular Risk Collaboration

Prof. Emanuele Angelantonio (Department of Public Health and Primary Care, University of Cambridge, Victor Phillip Dahdaleh Heart & Lung Research Institute), Prof. Ana Abreu (Hospital de Santa Maria, Faculty of Medicine, Lisbon), Prof. Frank Visseren (Department of Vascular Medicine, University Medical Center Utrecht), Prof. Maryam Kavousi (Department of Epidemiology Erasmus MC, University Medical Center Rotterdam), Prof. John William McEvoy (Preventive Cardiology, School of Medicine, University of Galway), Prof. Angela Wood (Department of Public Health and Primary Care, University of Cambridge, Victor Phillip Dahdaleh Heart & Lung Research Institute).

## Supplementary Material

ehag153_Supplementary_Data

## Declarations

### Data Availability

Data used for the current study are available upon reasonable request and approval of the individual data sources or collaborative groups (availability and methods may depend on the specific data source used). Data for the UCC-SMART cohort are available upon reasonable request after approval by the UCC-SMART Study group. Access to the data request module can be applied for via ucc-smart@umcutrecht.nl. Results are based on calculations by UMC Utrecht in project number [9851] using non-public microdata from Statistics Netherlands. The accompanying R code and a minimal reproducible example using synthetic data are available upon reasonable request to the corresponding author.

### Pre-registered Clinical Trial Number

None supplied.
