# Supplementary material for: Prediction of incident heart failure in established atherosclerotic cardiovascular disease: the SMART2-HF model
Source: Eur Heart J. 2026 Mar 11;47(34):4743–57. doi: 10.1093/eurheartj/ehag153 (PMC13549727; doi:10.1093/eurheartj/ehag153)
Supplement: Supplementary file 1 [file ehag153_supplementary_data.zip › REVISED2_Reitsma_SMART2-HF_Supplements.docx]

**SUPPLEMENTARY MATERIAL**
Supplement to: Prediction of incident heart failure in patients with established atherosclerotic cardiovascular disease: the SMART2-HF model.

Contents

[Supplementary Methods	2](#_Toc217302438)

[*External validation data sources*	2](#_Toc217302439)

[*Missing data*	4](#_Toc217302440)

[*Predictor selection*	4](#_Toc217302441)

[*Model development*	4](#_Toc217302442)

[*Lifetime prediction using lifetable method*	5](#_Toc217302443)

[*Recalibration*	6](#_Toc217302444)

[*Calibration plots*	7](#_Toc217302445)

[*Individual treatment benefit*	7](#_Toc217302446)

[Supplementary Tables	9](#_Toc217302447)

[*Table S1: Definition of ASCVD subtypes and HF at baseline*	9](#_Toc217302448)

[*Table S2: Outcome definition*	11](#_Toc217302449)

[*Table S3: Selected predictors*	12](#_Toc217302450)

[*Table S4: Missings of variables in derivation and validation data sources*	13](#_Toc217302451)

[*Table S5. Endpoint composition in the derivation cohort*	14](#_Toc217302452)

[*Table S6: Hazard ratios of the SMART2-HF model*	15](#_Toc217302453)

[*Table S7. Interval specific baseline survival for both outcomes*	16](#_Toc217302454)

[*Table S8. Equation for the SMART2-HF model*	17](#_Toc217302455)

[*Table S9. C-statistics of SMART2-HF compared to PREVENT-HF equations in external validation*	18](#_Toc217302456)

[*Table S10. Recalibration factors for the SMART2-HF model for European risk regions*	19](#_Toc217302457)

[*Table S11. Calibration diagnostics for incident HF in external validation*	20](#_Toc217302458)

[*Table S12. Sensitivity analysis in model derivation*	21](#_Toc217302459)

[Supplementary Figures	22](#_Toc217302460)

[*Figure S1: Flowchart of the study population in UCC-SMART cohort*	22](#_Toc217302461)

[*Figure S2. European risk regions based on standardized CVD mortality rates (From SCORE2, Hageman et al 2021^18^)*	23](#_Toc217302462)

[*Figure S3. Smoothing of baseline survival probabilities*	24](#_Toc217302463)

[*Figure S4. Internal model performance in UCC-SMART*	25](#_Toc217302464)

[*Figure S5. C-statistics in external validation stratified by sex*	26](#_Toc217302465)

[*Figure S6. C-statistics in external validation stratified by ASCVD type*	27](#_Toc217302466)

[*Figure S7. Calibration of incident HF in women*	28](#_Toc217302467)

[*Figure S8. Calibration of incident HF in men*	29](#_Toc217302468)

[*Figure S9. Calibration of non-HF mortality in external validation*	30](#_Toc217302469)

[*Figure S10. Decision curve analysis*	31](#_Toc217302470)

[*Figure S11. Comparison of external discrimination of alternative models in two external validation data sources (sensitivity analysis)*	32](#_Toc217302471)

[*Figure S12. Distribution of SMART2-HF and SMART2 predicted risks*	33](#_Toc217302472)

[*Figure S13. Estimation of individual risk of incident HF and individual treatment benefit*	34](#_Toc217302473)

[Supplementary References	35](#_Toc217302474)

**Supplementary Methods**

***External validation data sources***

The UK’s CPRD GOLD database^1^ is a repository for longitudinal individual primary care patient data collected from 1987 onwards. Over time, 966 primary care centres have contributed data for >20 million patients, with 407 practices actively contributing data for >3 million patients currently. For the most part CV disease prevention is undertaken in primary care and general practitioners are incentivized to use standard coding procedures to record this activity. For the current study, individuals with a history of CV disease between January 1st 2006-2008 were included.
*Flowchart CPRD validation population*

HUNT3^2^ is a population-based health study in the Nord-Trøndelag County in Norway conducted between 2006-2008. A total of 93,860 residents were contacted, of which 50,807 participated (54.1%). Information on history of ASCVD and HF was obtained via questionnaires and through linkage to hospital registries. Follow-up information was obtained by linkage to hospital and national registries for incident HF and non-HF death.

The SWEDEHEART (Swedish Web-system for Enhancement and Development of Evidence-based care in Heart disease Evaluated According to Recommended Therapies) registry^3^ is a nationwide registry that collects information on patient characteristics, in-hospital events, treatment and medications (at arrival, in-hospital, and discharge), and follow-up visits from all Swedish coronary care units with an almost complete coverage for patients with acute myocardial infarction and age below 80.^3^ For the current study, patients aged between 40-80 years old were included who had a follow-up visit 6-10 weeks following the discharge of a myocardial infarction event between February 9^th^ 2005 and February 1^st^ 2020 and that were free of heart failure at baseline. The follow-up visit at 6-10 weeks following discharge was taken as the baseline visit for risk estimation and follow-up started thereafter.

*Flowchart SWEDEHEART validation population:*

The ASCVD-PARTICLES cohort is part of the larger EP-PARTICLES study^4^, a comprehensive, population-based epidemiological project conducted in Eastern Poland. The primary objective of the study is to assess the impact of environmental risk factors, such as air pollution, ambient noise, light exposure, socio-economic deprivation, and climate change, on the incidence, progression, and mortality of atherosclerotic cardiovascular diseases (ASCVD). Patients in the ASCVD-PARTICLES cohort reside in the Podlaskie Voivodeship, a region notable for its high proportion of elderly individuals and prolonged exposure to airborne pollutants, especially fine particulate matter associated with the so-called “Polish smog.” At the same time, Podlaskie is one of the regions with the highest predicted life expectancy in Poland, which adds complexity to CV risk modelling. Patients were enrolled in this retrospective-prospective observational study between 2012 and 2016. The follow-up period extended until January 15, 2023. A hybrid follow-up strategy was applied, combining structured in hospital visits with event validation using official data sources: the Central Statistical Office (Olsztyn, Poland) and the National Health Fund (Białystok, Poland).

The Estonian Biobank is a population-based cohort maintained by the University of Tartu.^5^ It currently includes approximately 212,000 participants, representing roughly 20% of the Estonian adult population. Participants were recruited nationwide during two major enrolment waves (2002-2010 and 2018-2022) through primary care providers, hospitals, and dedicated recruitment offices. All individuals provided broad informed consent, donated blood samples, and completed a baseline questionnaire covering lifestyle, medical history, medication use, and demographic factors. Estonian Biobank is uniquely integrated with Estonia's national digital health system, allowing continuous linkage to electronic health records, including diagnoses, procedures, prescriptions, and mortality data. For the current study, we included individuals aged 40-80 years with available baseline data with established ASCVD and without a history of heart failure at baseline. Follow-up for outcomes was based on nationwide registry data, which provides near-complete coverage of health events in Estonia.

In the international, prospective REACH registry^6–9^, participants were enrolled between 2003 and 2004 from physician outpatient practices in several regions, including Western and Eastern Europe. Participants were followed for a planned follow-up of 2 years, some sites extended follow-up to 4 years. Incident heart failure was not continuously recorded; only visit-level yes/no information was available.^6–9^ Therefore, only discrimination was assessed in the REACH registry. Data on ASCVD diagnosis and history of HF were evaluated at baseline according to definitions used in the REACH registry.^6^ Data on clinical endpoints were collected through annual questionnaires and used the same definitions as for disease history data.

***Missing data***

Missing data in the derivation data (UCC-SMART cohort) is reported in Table S4. Missing data were imputed using the multivariate imputation by chained equations (R-package *mice)* with 5 imputations and 5 iterations. Missing data were considered to be missing at random (MAR). Both outcomes (incident HF and non-HF death) were included using their Nelson-Aalan estimators and event indicators. There was no missingness in outcome data. Continuous predictors were imputed with predictive mean matching and binary predictors were imputed with logistic regression. Transformations were included as just another variable in the imputation model. Convergence of the imputation model was assessed visually through the traces of the means of each imputed variable; consequently we used 5 iterations to ensure all imputation models had converged. Model coefficients were pooled using Rubin’s rules.^10^ Baseline hazards were calculated using these pooled coefficients in a random imputed dataset as there were no missing data for baseline age, follow-up time and events in UCC-SMART.
In external validation, missing data was handled by single imputation in ASCVD-Particles, HUNT3, CPRD and REACH. Missingness in SWEDEHEART and Estonian Biobank was handled by multiple imputation (also using 5 imputation sets, using the same imputation strategy as the derivation data). For validation in multiple imputed data, C-statistics were also pooled using Rubin’s rules.^10^ Pooled EO-ratios were used for model recalibration. For computational reasons, calibration plots were assessed in a random imputed dataset. In case of unavailability of predictors in external validation, median values of predictor values from derivation data were used.

***Predictor selection***

To allow synergistic use of the SMART2-HF score with the SMART2 score^11^, all predictors of the SMART2 model were included in the SMART2-HF model. On top of this, HF-specific predictors were added to the model. A pre-selection was made based by reviewing existing models for incident HF in this population, which resulted in the following candidate predictors: atrial fibrillation and body mass index. In subsequent discussions with an expert panel consisting of all clinical cardiologists and internists in the SMART2-HF study group, it was decided to include variables that are expected to be routinely available in this population and are of clear clinical relevance. Based on these criteria, both history of atrial fibrillation and body mass index were added to the SMART2-HF model.

***Model development***

The need for non-linear transformations for continuous predictors was investigated by comparing Akaike’s information criterium (AIC) of the model without transformations to models with, respectively, squared, or cubic polynomials, and logarithmic, or square root transformations. Schoenfeld residuals were assessed visually to assess the proportionality of the hazard-ratios. Interactions between age for specific predictors were added in case of violations of proportionality. Sex interactions were included for all predictors (excluding polynomials of continuous predictors) to account for sex-specific differences in relative effects of predictors. To reduce optimism, shrinkage was applied using Ridge regression, which uses a penalty parameter to shrink coefficients. The penalty parameter was estimated using 5-fold cross-validation. Ridge regression was applied using the optL2 function from the *penalized* package, using standardize = TRUE statement to scale all predictors before shrinkage. 95% confidence intervals for the penalized coefficients were obtained via bootstrapping with 1000 samples. Afterwards penalized coefficients were pooled across the 5 imputed datasets using Rubin’s rules.^10^ Baseline survival probabilities for 1-year age intervals were calculated across the entire age range of the derivation population (40-90 years). Baseline survival probabilities were estimated using the pooled coefficient for a reference patient in whom all binary predictors were set to 0, corresponding to the absence of the predictor and for sex the reference category (0) corresponded to a woman. The following levels for continuous predictors were used: age at baseline 40 years, BMI 25 kg/m^2^, eGFR 80 mL/min/1.73m^2^, CRP 2 mg/L, non-HDL 2.5 mmol/L, systolic blood pressure 120 mmHg, years since first ASCVD diagnosis 1 year. The baseline survival probabilities were then smoothed for both outcomes using a non-linear function weighted by the number of observations within each age interval. For both outcomes an exponential model (Nonlinear Least Squared) was used to smoothen the baseline survival probabilities.

***Lifetime prediction using lifetable method***

The lifetable method combines the baseline hazards with the coefficients from the cause-specific Cox models to calculate competing risk adjusted individual risk for incident HF and non-HF death. These are the steps for lifetime prediction using the lifetable method, with an example of a lifetable below.

An individual life-table includes the patient’s risk of incident HF (a_t_) and non-HF mortality (b_t_) for each 1 year age interval from the patient’s age at baseline up to the maximum age in the cohort (90 years). Next, interval-specific HF-free survival probability was calculated (p_t_ = 1 – a_t_ – b_t_) for each 1 year age interval. Cumulative HF-free survival probabilities were calculated by multiplying the survival probabilities of all previous intervals. Logically, the cumulative HF-free survival started at 100% at the current age of a person in the lifetable. Cumulative HF-free survival was calculated until the maximum age of 90 years. HF-free life expectancy was defined as the age where the estimated cumulative survival drops below 50% and can be read from the lifetable. Using this lifetable approach, the model could individually predict the any-year risk and lifetime risk of incident HF and non-HF mortality, the HF-free survival and the HF-free life expectancy in patients with ASCVD.

*Example of a lifetable:*

| **Age** | **Interval specific survival of incident HF (%)** | **Interval specific survival of non-HF death (%)** | **Interval specific event-free survival (%)** | **Cumulative event-free survival (%)** | **Interval specific risk of incident HF (%)** | **Interval specific risk non-HF death (%)** | **Cumulative risk of incident HF (%)** | **Cumulative risk of non-HF death (%)** |
| --- | --- | --- | --- | --- | --- | --- | --- | --- |
| 60 | 98.94723 | 99.72395 | 98.67118 | 100 | 1.052766 | 0.276053 | 0 | 0 |
| 61 | 98.81535 | 99.68043 | 98.49579 | 98.67118 | 1.168905 | 0.315321 | 1.0528 | 0.2761 |
| 62 | 98.66702 | 99.63004 | 98.29706 | 97.18696 | 1.29548 | 0.359553 | 2.2217 | 0.5914 |
| 63 | 98.50021 | 99.57168 | 98.07189 | 95.53192 | 1.432777 | 0.409187 | 3.5172 | 0.9509 |
| 64 | 98.31264 | 99.50407 | 97.81671 | 93.68996 | 1.580888 | 0.464638 | 4.9499 | 1.3601 |
| 65 | 98.10175 | 99.42574 | 97.5275 | 91.64443 | 1.739639 | 0.526273 | 6.5308 | 1.8248 |
| 66 | 97.86469 | 99.33499 | 97.19968 | 89.37852 | 1.908506 | 0.594378 | 8.2705 | 2.351 |
| 67 | 97.59826 | 99.2298 | 96.82807 | 86.87564 | 2.086526 | 0.669112 | 10.179 | 2.9454 |
| 68 | 97.29888 | 99.10787 | 96.40675 | 84.12 | 2.27218 | 0.750458 | 12.2655 | 3.6145 |
| 69 | 96.96256 | 98.96649 | 95.92904 | 81.09736 | 2.463285 | 0.838153 | 14.5377 | 4.365 |
| 70 | 96.58483 | 98.80249 | 95.38732 | 77.79592 | 2.656861 | 0.931617 | 17.001 | 5.2031 |
| 71 | 96.16073 | 98.61219 | 94.77292 | 74.20745 | 2.849021 | 1.029861 | 19.6578 | 6.1347 |
| 72 | 95.68473 | 98.39127 | 94.07601 | 70.32856 | 3.034866 | 1.131394 | 22.5068 | 7.1646 |
| 73 | 95.15068 | 98.13469 | 93.28537 | 66.16231 | 3.208424 | 1.234131 | 25.5417 | 8.296 |
| 74 | 94.55175 | 97.8365 | 92.38825 | 61.71975 | 3.362647 | 1.335305 | 28.7501 | 9.5301 |
| 75 | 93.88039 | 97.48971 | 91.37011 | 57.0218 | 3.48951 | 1.43141 | 32.1128 | 10.8654 |
| 76 | 93.12827 | 97.08607 | 90.21434 | 52.10088 | 3.580233 | 1.518181 | 35.6023 | 12.2968 |
| 77 | 92.28618 | 96.6158 | 88.90199 | 47.00247 | 3.625684 | 1.590656 | 39.1825 | 13.815 |
| 78 | 91.34405 | 96.06727 | 87.41131 | 41.78612 | 3.616988 | 1.643337 | 42.8082 | 15.4057 |
| 79 | 90.29081 | 95.42656 | 85.71737 | 36.5258 | 3.546358 | 1.670485 | 46.4252 | 17.049 |
| 80 | 89.11446 | 94.67697 | 83.79143 | 31.30896 | 3.408149 | 1.666585 | 49.9715 | 18.7195 |
| 81 | 87.80195 | 93.79827 | 81.60021 | 26.23422 | 3.200064 | 1.626977 | 53.3797 | 20.3861 |
| 82 | 86.33924 | 92.76575 | 79.10499 | 21.40718 | 2.924383 | 1.548649 | 56.5798 | 22.0131 |
| 83 | 84.71134 | 91.54898 | 76.26031 | 16.93415 | 2.589005 | 1.431109 | 59.5041 | 23.5617 |
| 84 | 82.90234 | 90.10994 | 73.01228 | 12.91404 | 2.207997 | 1.277206 | 62.0931 | 24.9928 |
| 85 | 80.89562 | 88.40046 | 69.29608 | 9.428832 | 1.80132 | 1.093701 | 64.3011 | 26.27 |
| 86 | 78.674 | 86.35826 | 65.03226 | 6.533811 | 1.3934 | 0.891325 | 66.1025 | 27.3637 |
| 87 | 76.2201 | 83.90084 | 60.12095 | 4.249085 | 1.010428 | 0.684067 | 67.4959 | 28.2551 |
| 88 | 73.51678 | 80.91531 | 54.43209 | 2.55459 | 0.676538 | 0.487536 | 68.5063 | 28.9391 |
| 89 | 70.54773 | 77.24033 | 47.78807 | 1.390517 | 0.409539 | 0.316477 | 69.1828 | 29.4267 |
| 90 |  |  |  | 0.664501 |  |  | 69.5924 | 29.7431 |

Risk predictor profile: a 60-year-old woman from low-risk region, CAD = 1, CeVD = 0, PAD = 0, AAA = 0, Diabetes = 0, Atrial fibrillation = 1, Current smoker = 0, BMI = 30kg/m^2^, SBP = 150 mmHg, CRP = 5 mg/L, eGFR = 70 mL/min/1.73 m², non-HDL = 2.0 mmol/L, yearsSinceFirstDiagnosis = 2 years. See Table S8 for the equation of SMART2-HF model.

***Recalibration***

The SMART2-HF model was recalibrated to the low, moderate and high European risk regions using data from representative data sources from those regions (CPRD for the low-risk region, SWEDEHEART for the moderate-risk region, ASCVD-Particles for the high-risk). Recalibration required the sex-specific expected-observed (EO) ratio’s for both outcomes in these cohorts. The process involves the following steps:

1. Using the SMART2-HF model the predicted risk of incident HF and non-HF death at 10 years (for ASCVD-Particles this was at 9 years) was estimated. The mean predicted risk for men and women separately was calculated. The SMART2-HF model provides predictions up to age 90. For example, only patients aged 80 years or younger have a 10-year predicted risk.

2. The observed cumulative incidence for men and women separately was estimated for the same patients who have a predicted risk at the selected time horizon. This can be done by using the *cuminc* function in R.

3. The sex-specific EO ratios were calculated by dividing the sex-specific mean predicted risk (Step 1) by the sex-specific observed cumulative incidence (Step 2).

4. The SMART2-HF model was recalibrated by subtracting the natural logarithm of the sex-specific EO ratio from the linear predictor for both outcomes (LP – ln(EO)) in all representative data sources. The sex-specific EO ratios for the low, moderate and high European risk regions are presented in Supplementary Table S10.

***Calibration plots***

Calibration plots were made accounting for competing risks. First, predicted risks were ordered from low to high. Risks were rounded to no decimals (e.g., 38.1% was rounded to 38%) and all unique predicted risks in the data were extracted. At each of the unique risk estimates the number of observed events was estimated using the *cuminc* function R, obtaining both the observed incidence at that particular risk estimate as well as the standard error for the estimation. Following sequencing along all predicted risks a loess regression was performed using the number of observations at each unique risk as weights. Similarly, the confidence intervals were obtained by using the standard errors to obtain 95% confidence intervals, to which the same loess regression was applied to obtain smoothed estimates. As some unique risks have few observations a dynamic window was used that included the observations from the unique risk estimate and those within ±1% from that unique risk. This window was expanded dynamically (by multiplying the window of 1% with 1.5 etc.) until it contained at least two primary events and two other outcomes (either competing events or censored cases). For visualization purposes, the predicted risks were truncated at the 99^th^ percentile, or 95^th^ percentile for smaller data sources, to avoid unstable estimates in regions with very few individuals. The background histogram in the plots (in blue) shows the distribution of predicted risks in the population, allowing assessment of the density of predictions across different risk levels.

***Individual treatment benefit***

It has been previously illustrated how individual survival estimates can be combined with treatment effects (i.e. hazard ratios) from trials to estimate individual absolute risk reduction from treatment.^12–14^ The HRs can be combined with the lifetable estimation by adjusting the linear predictor as follows: LP_with treatment_ = LP_current treatment_ + ln(HR)

For these analyses, hazard ratios derived from trials are assumed to be proportional over time. Estimating the effect of more than one therapy, the HR’s for both therapies should be multiplied and are assumed independent.

These benefits can be expressed as individual absolute risk reductions over any time horizon as well as gain in HF-free life years due to treatment. The individual absolute risk reduction is calculated as the difference between the predicted risk on current treatment and the new treatment regimen. Similarly, the individual lifetime benefit is calculated by taking the difference between the predicted event-free life expectancy with and without a certain treatment.
For illustration, an individual treatment benefit of lowering systolic blood pressure with 20 mmHg based on a meta-analysis^15^ (hazard ratio (HR) 0.72 (95%CI 0.67-0.78) per 10 mmHg blood pressure lowering for incident HF and HR 0.87 (95% 0.84-0.91 per 10 mmHg blood pressure lowering for non-HF death) was estimated for two example patients, shown in Figure S13. Uncertainty was determined by sampling (n=1000) from a log-normal distribution following the 95% CI from this meta-analysis.

*Integrating treatment effects for patients on current treatment*

If a patient is already receiving one or more evidence-based treatments, the corresponding treatment effects can be incorporated into the SMART2-HF model in the same way. By applying the corresponding hazard ratios for the current treatment to the predicted risks, the risk estimates reflect the patient’s current treatment regimen.

**Supplementary Tables**

**Table S1:** Definition of ASCVD subtypes and HF at baseline

| Data source | Definition of ASCVD and HF at baseline and incident HF at follow-up | |
| --- | --- | --- |
| UCC-SMART | *Evaluation*: Data on ASCVD diagnosis is evaluated by a physician at baseline according to the following definitions. History of HF was not documented at baseline and was extracted by linking with the Dutch Hospital Data and defined by ICD-9 and ICD-10 codes. Incident HF was not an adjudicated endpoint in this cohort and was also extracted by linkage with Dutch Hospital Data. | |
|  | *Coronary artery disease* | Angina pectoris with documented stenosis, myocardial infarction, or coronary revascularization (coronary bypass surgery or coronary angioplasty), based on questionnaire at inclusion, and/or previous hospitalization with the following ICD-10-codes:  I20-I25. |
|  | *Cerebrovascular disease* | Transient ischaemic attack, cerebral infarction, amaurosis fugax or retinal infarction, or a history of carotid surgery and/or previous hospitalization with the following ICD-10-codes: I60-69, G45. |
|  | *Peripheral artery disease* | Symptomatic and documented obstruction of distal arteries of the leg or a history of vascular surgery of the leg (percutaneous transluminal angioplasty, bypass, or amputation) and/or previous hospitalization with the following ICD-10-codes: I70.0-I70.8. |
|  | *Abdominal aortic aneurysm* | Supra- or infrarenal aneurysm of the aorta (distal aortic anteroposterior diameter ≥3 cm, measured at baseline examination with ultrasonography) or a history of AAA surgery and/or previous hospitalization with the following ICD-10-codes: I71.3-I71.9 |
|  | *History of HF* | Hospitalization with ICD-10 codes (as described in Table S2) registered at any diagnostic position in hospital records prior to baseline date (history) or during follow-up (incident). |
| CPRD, ASCVD-Particles,  SWEDEHEART,  Estonian Biobank | *Evaluation:* Data on ASCVD diagnosis, history of HF and incident HF were defined using ICD-10 codes registered in hospital and national registries. | |
|  | *Coronary artery disease* | Previous hospitalization with following ICD-10 codes: I20-I25 |
|  | *Cerebrovascular disease* | Previous hospitalization with following ICD-10 codes: I60-69, G45 |
|  | *Peripheral artery disease* | Previous hospitalization with following ICD-10 codes: I70.0-I70.8 |
|  | *Abdominal aortic aneurysm* | Previous hospitalization with following ICD-10 codes: I71.3-I71.9 |
|  | *History of HF and incident HF* | Hospitalization with ICD-10 codes (as described in Table S2) registered at any diagnostic position in hospital records prior to baseline date (history) or during follow-up (incident). |
| HUNT3 | Evaluation: Data on ASCVD diagnosis, history of HF and incident HF are defined using ICD-9 and ICD-10 codes registered in hospital and national registries. | |
|  | *Coronary artery disease* | ICD-9: 410, 411, 412, 413, 414, 429.2, 429.7, V45.81, V45.82. ICD-10: I20-I25, Z95.1, Z95.5, Z98.61 |
|  | *Cerebrovascular disease* | ICD-9: 362.34, 430-438. ICD-10: I60-69, G45, G46, H34.0 |
|  | *Peripheral artery disease* | ICD-9: 440, 441, 443.89, 443.9, 444, 447.1, 557.1, 557.9, V43.4. ICD-10: I70, I71, I73.8, I73.9, I74, I77.1, I79.0, K55.1, K55.8, K55.9, Z95.82, Z98.62 |
|  | *Abdominal aortic aneurysm* | No information gathered. |
|  | *History of HF and incident HF* | Hospitalization with ICD-10 codes (as described in Table S2) registered at any diagnostic position in hospital records prior to baseline date (history) or during follow-up (incident). |
| REACH registry | *Evaluation*: Data on ASCVD diagnosis, history of HF and incident HF were evaluated according to the following definitions. | |
|  | *Coronary artery disease* | Stable angina with documented coronary artery disease, history of unstable angina with documented coronary artery disease, history of percutaneous coronary intervention, history of coronary artery bypass graft surgery, or previous myocardial infarction. |
|  | *Cerebrovascular disease* | Hospital or neurologist report with the  diagnosis of TIA or ischemic stroke. |
|  | *Peripheral artery disease* | One or both of the following criteria: Current intermittent claudication with ankle-brachial index of <0.9 and/or history of intermittent claudication together with previous related intervention (angioplasty, bypass graft, and other vascular intervention including amputation). |
|  | *Abdominal aortic aneurysm* | No information gathered. |
|  | *History of HF and incident HF* | The presence of signs and symptoms of either right or left ventricular failure or both and the diagnosis should be confirmed by non-invasive or hemodynamic measurements. |

**Table S2:** Outcome definition

| Endpoints included | ICD-10 codes |
| --- | --- |
| Fatal and non-fatal heart failure |  |
| Hypertensive heart disease with (congestive) heart failure | I11.0 |
| Hypertensive heart and renal disease with (congestive) heart failure | I13.0 |
| Hypertensive heart and renal disease with both (congestive) heart failure and renal failure | I13.2 |
| Ischaemic cardiomyopathy | I25.5 |
| Dilated cardiomyopathy | I42.0 |
| Other restrictive cardiomyopathy | I42.5 |
| Other cardiomyopathies | I42.8 |
| Cardiomyopathy, unspecified | I42.9 |
| Cardiomyopathy in diseases classified elsewhere | I43 |
| Heart Failure | I50 |
| Pulmonary oedema | J81 |
| Chronic passive congestion of liver | K76.1 |
| Cardiogenic shock | R57.0 |

Above ICD-10 codes were used to identify hospitalizations with heart failure as any diagnosis at discharge (non-fatal heart failure) and deaths with heart failure as primary or secondary cause of death (fatal heart failure). Endpoint definitions depend on data source availability but where ideally defined as stated above.

**Table S3:** Selected predictors

a. SMART2 predictors (n=13)

To allow synergistic use of the SMART2-HF model with the SMART2 model^11^, all predictors of the SMART2 model were included.

| Demographic | Medical history | Physical signs | Laboratory tests |
| --- | --- | --- | --- |
| Age | Current smoking | Systolic blood pressure | Non-HDL cholesterol |
| Sex | Diabetes mellitus |  | Estimated GFR |
|  | Presence of CAD |  | CRP |
|  | Presence of CeVD |  |  |
|  | Presence of PAD |  |  |
|  | Presence of AAA |  |  |
|  | Years since first ASCVD diagnosis |  |  |

b. All predictors (n=15)

Two predictors (in blue) were added by the expert panel based on clinical relevance in the development of incident HF and clinical availability.

| Demographic | Medical history | Physical signs | Laboratory tests |
| --- | --- | --- | --- |
| Age | Current smoking | Systolic blood pressure | Non-HDL cholesterol |
| Sex | Diabetes mellitus | BMI | Estimated GFR |
|  | Presence of CAD |  | CRP |
|  | Presence of CeVD |  |  |
|  | Presence of PAD |  |  |
|  | Presence of AAA |  |  |
|  | Years since first ASCVD diagnosis |  |  |
|  | Atrial fibrillation |  |  |

Abbreviations: CAD = coronary artery disease; CeVD = cerebrovascular disease; PAD = peripheral artery disease; AAA = abdominal aortic aneurysm; ASCVD = atherosclerotic cardiovascular disease; non-HDL = high-density lipoprotein; GFR = glomerular filtration rate (based on creatinine, calculated with CKD-EPI formula^16^); CRP = c-reactive protein; BMI = body mass index.

|  | Missings (%) | | | | | | |
| --- | --- | --- | --- | --- | --- | --- | --- |
|  | *Derivation* | *External validation* | | | | | |
|  | UCC-SMART | CPRD | HUNT3 | SWEDEHEART | Estonian Biobank | ASCVD -Particles | REACH |
| Age | 0 | 0 | 0 | 0 | 0 | 0 | 0 |
| Sex | 0 | 0 | 0 | 0 | 0 | 0 | 0 |
| Current smokers | 0.4 | 41 | 0 | 0.2 | 22.1 | 0 | 2.5 |
| Body mass index | 0.3 | 50 | 1.7 | 7.5 | 0.5 | 0 | 1.1 |
| Systolic blood pressure | 0.2 | 6 | 1.3 | 9.5 | 9.6 | 0 | 0.2 |
| Non-HDL cholesterol^a^ | 0.5 | 31 | 2.8 | 18.7 | * | 5.2 |  |
| *Total cholesterol* |  |  |  |  |  |  | 19 |
| *HDL cholesterol* |  |  |  |  |  |  | * |
| Estimated GFR | 0.3 | 17 | 1.7 | 2.7 | 61.7 | 2.0 | 22.9 |
| CRP | 12.0 | 90 | 1.7 | 18.2 | 53 | * | * |
| Diabetes mellitus | 0 | 0 | 0 | 0 | 0 | 0 | 0.5 |
| Presence of CAD | 0 | 0 | 0 | 0 | 0 | 0 | 0 |
| Presence of CeVD | 0 | 0 | 0 | 0 | 0 | 0 | 0 |
| Presence of PAD | 0 | 0 | 0 | 0 | 0 | 0 | 0 |
| Presence of AAA | 0 | 0 | * | 0 | 0 | 0 | 7.6 |
| Atrial fibrillation | 0 | * | 0 | 0 | 0 | 0 | 0.8 |
| Years since first ASCVD diagnosis | 1.0 | 0 | 0 | 0 | 0 | 0 | 0 |

**Table S4:** Missings of variables in derivation and validation data sources

* Predictor value is missing completely in this external validation data source and was imputed using sex-specific median imputation from the derivation population.^17^
^a^ For non-HDL, in case only one of the components was missing (e.g. total cholesterol and HDL cholesterol), this information was used to calculate non-HDL cholesterol. Abbreviations: non-HDL = non high-density lipoprotein; GFR = glomerular filtration rate (based on creatinine, calculated with CKD-EPI formula^16^); CRP = c-reactive protein; CAD = coronary artery disease; CeVD = cerebrovascular disease; PAD = peripheral artery disease; AAA = abdominal aortic aneurysm; ASCVD = atherosclerotic cardiovascular disease.

## Table S5. Endpoint composition in the derivation cohort

| *Composition of the endpoint* | N (%) |
| --- | --- |
| Hospitalization with HF | 991 (96%) |
| Primary diagnostic position | 586 (59%) |
| Secondary diagnostic positions | 405 (41%) |
| HF death | 40 (4%) |
| **Total incident HF events** | **1,031** |
|  |  |
| *Preceding events prior to HF* | N (%) |
| Recurrent CV events | 154 (15%) |
| Myocardial infarction | 103 (67%) |
| **Total incident HF events** | **1,031** |

**Table S6:** Hazard ratios of the SMART2-HF model

|  | **Hazard ratio (95% CI)** | |
| --- | --- | --- |
|  | Incident HF | non-HF death |
| Age as time-axis | * | * |
| *Main effects* |  |  |
| Baseline age (in years) | 0.94 (0.93 - 0.96) | 0.94 (0.92 - 0.95) |
| Male sex intercept^**^ | 1.55 (0.69 - 3.51) | 0.66 (0.14 - 3.09) |
| Coronary artery disease | 1.45 (1.05 – 2.00) | 1.16 (0.87 - 1.56) |
| Cerebrovascular disease | 1.16 (0.85 - 1.59) | 1.39 (1.05 - 1.84) |
| Peripheral artery disease | 1.34 (0.75 - 2.40) | 1.33 (0.83 - 2.14) |
| Abdominal aortic aneurysm | 1.32 (0.94 - 1.86) | 1.66 (1.25 - 2.21) |
| Atrial fibrillation | 2.37 (1.48 - 3.80) | 1.25 (0.82 - 1.91) |
| Diabetes mellitus | 1.83 (1.38 - 2.43) | 1.78 (1.45 - 2.18) |
| Current smoking | 1.38 (1.05 - 1.81) | 1.81 (1.47 - 2.22) |
| SBP - untransformed effect (in mmHg) | 0.99 (0.97 - 1.01) | 1.00 (1.00 – 1.00) |
| SBP - squared effect | 1.00 (1.00 – 1.00) |  |
| BMI - untransformed effect (in kg/m^2^) | 1.04 (1.02 - 1.07) | 0.32 (0.09 - 1.15) |
| BMI - squared effect |  | 1.03 (0.99 - 1.08) |
| BMI - cubic effect |  | 1.00 (1.00 – 1.00) |
| CRP - untransformed effect (in mg/L) | 1.06 (1.03 - 1.09) |  |
| CRP - square root effect |  | 1.26 (1.13 - 1.41) |
| Estimated GFR - untransformed effect (in mL/min/1.73m^2^) | 0.97 (0.95 – 1.00) | 0.93 (0.91 - 0.95) |
| Estimated GFR - squared effect | 1.00 (1.00 – 1.00) | 1.00 (1.00 – 1.00) |
| Non-HDL cholesterol (in mmol/L) | 0.97 (0.89 - 1.06) | 0.93 (0.86 - 1.01) |
| Years since first ASCVD diagnosis - square root effect (in years) | 1.14 (1.05 - 1.23) | 1.09 (1.02 - 1.16) |
| *Sex-interaction effects – applied for men* |  |  |
| Male sex*Baseline age (in years) | 1.00 (0.99 - 1.01) | 1.00 (0.99 - 1.02) |
| Male sex*Coronary artery disease | 0.92 (0.64 - 1.31) | 0.77 (0.55 - 1.07) |
| Male sex*Cerebrovascular disease | 1.07 (0.75 - 1.53) | 0.87 (0.64 - 1.19) |
| Male sex*Peripheral artery disease | 0.90 (0.48 - 1.67) | 0.93 (0.56 - 1.53) |
| Male sex*Abdominal aortic aneurysm | 1.08 (0.74 - 1.58) | 0.75 (0.55 - 1.04) |
| Male sex*Atrial fibrillation | 0.86 (0.52 - 1.44) | 1.01 (0.63 - 1.61) |
| Male sex*Diabetes mellitus | 0.86 (0.62 - 1.18) | 0.83 (0.65 - 1.06) |
| Male sex*Current smoking | 1.01 (0.74 - 1.37) | 0.90 (0.71 - 1.15) |
| Male sex*SBP - untransformed effect (in mmHg) | 1.00 (1.00 – 1.00) | 1.00 (1.00 - 1.01) |
| Male sex*BMI - untransformed effect (in kg/m^2^) | 1.00 (0.98 - 1.03) | 1.02 (0.99 - 1.04) |
| Male sex*CRP - untransformed effect (in mg/L) | 1.00 (0.96 - 1.03) |  |
| Male sex*CRP - square root effect (in mg/L) |  | 0.95 (0.83 - 1.08) |
| Male sex*Estimated GFR - untransformed effect (in mL/min/1.73m^2^) | 1.00 (0.99 - 1.01) | 1.00 (0.99 - 1.01) |
| Male sex*Non-HDL cholesterol (in mmol/L) | 0.99 (0.89 - 1.09) | 1.03 (0.94 - 1.14) |
| Male sex*Years since first ASCVD diagnosis - square root effect (in years) | 1.04 (0.95 - 1.14) | 1.01 (0.94 - 1.09) |
| Hazard ratios were calculated by exponentiating the model coefficients (found in the equation in Table S8). Coefficients were penalized through ridge regression; therefore, the reported hazard ratios should not be interpreted causally. 95% CI's were obtained via bootstrapping with 1000 samples. Sex interactions were included for all predictors (excluding polynomials of continuous predictors). Abbreviations: HF = heart failure, CI = confidence interval; SBP = systolic blood pressure; BMI = body mass index; CRP = C-reactive protein; GFR = glomerular filtration rate (calculated with CKD-EPI formula 2009); HDL = high-density lipoprotein; ASCVD = atherosclerotic cardiovascular disease.* Age effects are also captured in the baseline hazard of the model as age was used as the underlying time-axis. ** Sex effects are also captured in the sex-interactions effects. | | |

**Table S7.** Interval specific baseline survival for both outcomes

| Age | 1-year survival for incident HF | 1-year survival for non-HF death |
| --- | --- | --- |
| 40 | 0.999642976 | 0.999629396 |
| 41 | 0.999598081 | 0.999571117 |
| 42 | 0.99954754 | 0.999503674 |
| 43 | 0.999490643 | 0.999425625 |
| 44 | 0.999426592 | 0.999335303 |
| 45 | 0.999354487 | 0.999230777 |
| 46 | 0.999273314 | 0.999109814 |
| 47 | 0.999181935 | 0.998969829 |
| 48 | 0.999079064 | 0.998807831 |
| 49 | 0.998963257 | 0.998620359 |
| 50 | 0.998832888 | 0.998403405 |
| 51 | 0.998686125 | 0.998152335 |
| 52 | 0.998520907 | 0.997861784 |
| 53 | 0.998334913 | 0.997525542 |
| 54 | 0.99812553 | 0.997136425 |
| 55 | 0.997889818 | 0.996686118 |
| 56 | 0.997624465 | 0.996164999 |
| 57 | 0.997325745 | 0.995561932 |
| 58 | 0.99698946 | 0.994864031 |
| 59 | 0.996610888 | 0.994056383 |
| 60 | 0.996184712 | 0.993121729 |
| 61 | 0.995704944 | 0.992040098 |
| 62 | 0.995164846 | 0.990788376 |
| 63 | 0.994556831 | 0.989339817 |
| 64 | 0.993872359 | 0.987663467 |
| 65 | 0.993101816 | 0.985723506 |
| 66 | 0.992234377 | 0.983478479 |
| 67 | 0.99125786 | 0.980880414 |
| 68 | 0.990158546 | 0.977873794 |
| 69 | 0.988920996 | 0.974394374 |
| 70 | 0.987527824 | 0.970367803 |
| 71 | 0.985959463 | 0.965708039 |
| 72 | 0.984193883 | 0.960315513 |
| 73 | 0.982206283 | 0.954074993 |
| 74 | 0.979968745 | 0.946853131 |
| 75 | 0.977449839 | 0.938495606 |
| 76 | 0.974614183 | 0.928823832 |
| 77 | 0.971421948 | 0.917631139 |
| 78 | 0.967828292 | 0.904678357 |
| 79 | 0.963782739 | 0.889688707 |
| 80 | 0.959228463 | 0.872341883 |
| 81 | 0.954101492 | 0.852267213 |
| 82 | 0.94832981 | 0.829035733 |
| 83 | 0.941832347 | 0.802151025 |
| 84 | 0.934517835 | 0.771038605 |
| 85 | 0.926283533 | 0.735033652 |
| 86 | 0.917013777 | 0.693366799 |
| 87 | 0.90657836 | 0.645147692 |
| 88 | 0.894830702 | 0.589345967 |
| 89 | 0.881605791 | 0.524769233 |
| Values represent the probability of surviving the 1-year interval without experiencing the respective outcome, not accounting for the other outcome. Abbreviations: HF = heart failure. | | |

## **Table S8.** Equation for the SMART2-HF model

| **Linear predictor (LP) for incident HF**  1-year risk = 1 – (age-specific 1-year baseline survival^a^)^exp(LP_HF_) | |
| --- | --- |
| LP_HF_= | Main effects:  -0.05977633228*(age_baseline – 40) + 0.43965640220 (if men) + 0.36888923233 (if CAD) + 0.15046794864 (if CeVD) + 0.29251233672 (if AAA) + 0.27903012081 (if PAD) + 0.86324307854 (if atrial fibrillation) + 0.60476976686 (if diabetes mellitus) + 0.32300574635 (if current smoker) - 0.00868589824*(SBP – 120) + 0.00005578538*(SBP^2 – 120^2) + 0.04112123662*(BMI – 25) + 0.05780473151*(CRP – 2) - 0.02571108205*(eGFR – 80) + 0.00014837565*(eGFR^2 – 80^2) - 0.02897085577*(nonHDL – 2.5) + 0.12789339933*(√yearsSinceFirstDiagnosis – √1) – ln(1.058760, if women) – ln(1.162733, if men) - ln(sex-specific regional recalibration factor for incident HF^b^)    Sex interaction effects (only applied for men):  - 0.00310421359*(age_baseline – 40) - 0.08498086557 (if CAD) + 0.07033136995 (if CeVD) - 0.10635499929 (if AAA) + 0.07687946225 (if PAD) - 0.14896318158 (if atrial fibrillation) - 0.15063739850 (if diabetes mellitus) + 0.00818554029 (if current smoker) - 0.00057490554*(SBP – 120) + 0.00421634986*(BMI – 25) - 0.00384110953*(CRP – 2) + 0.00078783337*(eGFR – 80) - 0. 01243996683*(nonHDL – 2.5) + 0.03886565647*(√yearsSinceFirstDiagnosis – √1) |
| **Linear predictor (LP) for non-HF death**  1-year risk = 1 – (age-specific 1-year baseline survival^a^)^exp(LP_nHFD_) | |
| LP_nHFD_ = | Main effects:  -0.0653838402*(age_baseline – 40) - 0.4194928407 (if men) + 0.1494150141 (if CAD) + 0.3266254671 (if CeVD) + 0.2868356598 (if AAA) + 0.5086732620 (if PAD) + 0.2267524307 (if atrial fibrillation) + 0.5760046495 (if diabetes mellitus) + 0.5921479032 (if current smoker) + 0.0002715753 *(SBP – 120) - 1.1446257638 *(BMI – 25) + 0.0336027308*(BMI^2 – 25^2) -0.0003179087*(BMI^3 – 25^3) + 0.2350109386*(√CRP – √2) - 0.0732018151*(eGFR – 80) + 0.0004918357*(eGFR^2 – 80^2) - 0.0683972921*(nonHDL – 2.5) + 0.0854840849*(√yearsSinceFirstDiagnosis – √1) – ln(1.239799, if women) – ln(0.527330, if men) - ln(sex-specific regional recalibration factor for non-HF death^b^)  Sex interaction effects (only applied for men):  + 0.0036795060*(age_baseline – 40) - 0.2611792281 (if CAD) - 0.1398162872 (if CeVD) -0.0774947410 (if AAA) - 0.2846371406 (if PAD) + 0.0065394527 (if atrial fibrillation) - 0.1856422608 (if diabetes mellitus) - 0.1032600497 (if current smoker) + 0.0028484465*(SBP – 120) + 0.0161118750*(BMI – 25) - 0.0526413602*(√CRP – √2) - 0.0014473590*(eGFR – 80) + 0.0342276690*(nonHDL – 2.5) + 0.0121230631*(√yearsSinceFirstDiagnosis – √1) |
| ^a^ Age-specific 1-year baseline survivals are stated in Table S7.  ^b^ Sex-specific regional recalibration factors for both outcomes are stated in Table S10. Age_baseline in years at the moment of prediction; SBP: systolic blood pressure in mmHg; BMI: body mass index in kg/m^2^; eGFR: estimated glomerular filtration rate in mL/min/1.73 m^2^; CRP: C-reactive protein in mg/L; nonHDL: non high-density lipoprotein cholesterol in mmol/L; yearsSinceFirstDiagnosis in years. Abbreviations: HF = heart failure. | |

## Table S9. C-statistics of SMART2-HF compared to PREVENT-HF equations in external validation

| **Data source** | **N^*^** | **Events** | **Model** | **C-statistic (95% CI)** | **ΔC-statistic (95% CI)** |
| --- | --- | --- | --- | --- | --- |
| CPRD | 105,332 | 11,971 | *PREVENT-HF* | 0.663 (0.663 – 0.664) | *ref* |
|  |  |  | *SMART2-HF* | 0.682 (0.681 – 0.683) | 0.018 (0.018,0.019) |
| HUNT3 | 4,350 | 448 | *PREVENT-HF* | 0.711 (0.687 – 0.735) | *ref* |
|  |  |  | *SMART2-HF* | 0.732 (0.709 – 0.755) | 0.021 (0.002,0.040) |
| SWEDEHEART | 75,282 | 6,652 | *PREVENT-HF* | 0.706 (0.704 – 0.708) | *ref* |
|  |  |  | *SMART2-HF* | 0.721 (0.718 – 0.723) | 0.015 (0.013,0.016) |
| ASCVD-Particles | 1,435 | 170 | *PREVENT-HF* | 0.656 (0.610 – 0.702) | *ref* |
|  |  |  | *SMART2-HF* | 0.702 (0.657 – 0.747) | 0.046 (0.014,0.078) |
| Estonian Biobank | 9,145 | 893 | *PREVENT-HF* | 0.700 (0.683 – 0.718) | *ref* |
|  |  |  | *SMART2-HF* | 0.712 (0.692 – 0.732) | 0.012 (-0.007,0.031) |
| C-statistics were calculated for 10-year risks of incident HF using the Score() function (contrasts = TRUE). Difference in C-statistics was calculated with the PREVENT-HF model as reference. * Numbers can differ from those in Table 2, as PREVENT-HF equations cannot be calculated for 80-year olds and were therefore removed for this analysis.  Abbreviations: N = observations; CI = confidence interval. | | | | | |

**Table S10.** Recalibration factors for the SMART2-HF model for European risk regions

|  |  | European risk regions | | |
| --- | --- | --- | --- | --- |
|  |  | Low risk | Moderate risk | High risk |
| Women | Incident HF | 0.693124 | 0.457197 | 0.531005 |
|  | Non-HF death | 0.937189 | 0.896024 | 0.726944 |
| Men | Incident HF | 0.673257 | 0.535317 | 0.456008 |
|  | Non-HF death | 0.927437 | 0.974604 | 0.614254 |

## Table S11. Calibration diagnostics for incident HF in external validation

|  |  |  | | **EO ratios ± SD** | |
| --- | --- | --- | --- | --- | --- |
| **Data source** | **Intercept (95% CI)** | | **Slope (95% CI)** | *Women* | *Men* |
| EU - Low risk |  | |  |  |  |
| CPRD | 0.015 (-0.005, 0.034) | | 0.986 (0.950, 1.023) | 1.003 ± 0.002 | 0.982 ± 0.002 |
| HUNT3 | -0.415 (-0.518, -0.312) | | 0.779 (0.645, 0.914) | 1.349 ± 0.007 | 1.459 ± 0.006 |
| EU - Moderate risk |  | |  |  |  |
| SWEDEHEART | 0.007 (-0.022, 0.037) | | 0.950 (0.909, 0.991) | 0.997 ± 0.004 | 0.997 ± 0.002 |
| EU - High risk |  | |  |  |  |
| ASCVD-Particles | -0.034 (-0.208, 0.140) | | 0.801 (0.567, 1.035) | 0.998 ± 0.014 | 0.994 ± 0.015 |
| Estonian Biobank | -0.153 (-0.231, -0.074) | | 0.676 (0.595, 0.757) | 1.023 ± 0.004 | 0.984 ± 0.006 |
| Calibration intercept, calibration slope and sex-specific EO ratios for the outcome of incident HF in all external validation data sources where calibration was assessed. Abbreviations: EU = Europe; CI = confidence interval; EO ratio = expected-observed ratio; SD = standard deviation. | | | | | |

## Table S12. Sensitivity analysis in model derivation

|  | Original hazard ratios | Sensitivity analysis I | Sensitivity analysis II | Sensitivity analysis III |  |
| --- | --- | --- | --- | --- | --- |
|  |  | *Excluding events* ≤*6 months* | *Excluding HF death* | *Censoring for interim MI* |  |
| Incident HF events | 1,031 | 1,008 | 991 | 928 |  |
| Age | 0.94 | 0.94 | 0.94 | 0.95 |  |
| Age * Male sex | 0.99 | 0.99 | 0.99 | 1.00 |  |
| Abdominal aortic aneumrys | 1.45 | 1.49 | 1.41 | 1.64 |  |
| BMI | 1.05 | 1.04 | 1.05 | 1.05 |  |
| Cerebrovascular disease | 1.26 | 1.26 | 1.28 | 1.17 |  |
| Coronary artery disease | 1.60 | 1.61 | 1.60 | 1.57 |  |
| CRP | 1.07 | 1.07 | 1.07 | 1.06 |  |
| Diabetes mellitus | 1.90 | 1.87 | 1.92 | 1.77 |  |
| eGFR | 0.95 | 0.95 | 0.95 | 0.95 |  |
| eGFR - squared term | 1.00 | 1.00 | 1.00 | 1.00 |  |
| Atrial fibrillation | 2.48 | 2.54 | 2.57 | 2.62 |  |
| Current smoking | 1.48 | 1.47 | 1.44 | 1.48 |  |
| non-HDL cholesterol | 0.99 | 0.98 | 1.00 | 0.98 |  |
| Peripheral artery disease | 1.38 | 1.38 | 1.47 | 1.37 |  |
| SBP | 0.97 | 0.97 | 0.96 | 0.97 |  |
| SBP - squared term | 1.00 | 1.00 | 1.00 | 1.00 |  |
| Years since first ASCVD diagnosis - square root term | 1.12 | 1.11 | 1.11 | 1.10 |  |
| Male sex | 3.82 | 3.46 | 3.78 | 3.22 |  |
| Male sex * AAA | 0.82 | 0.81 | 0.86 | 0.70 |  |
| Male sex | 1.00 | 1.00 | 1.00 | 1.00 |  |
| Male sex * CeVD | 0.99 | 1.00 | 0.98 | 1.10 |  |
| Male sex * CAD | 0.81 | 0.80 | 0.83 | 0.80 |  |
| Male sex * CRP | 0.99 | 0.99 | 0.99 | 0.99 |  |
| Male sex * Diabetes mellitus | 0.83 | 0.84 | 0.83 | 0.88 |  |
| Male sex * eGFR | 1.00 | 1.00 | 1.00 | 1.00 |  |
| Male sex * Atrial fibrillation | 0.83 | 0.77 | 0.79 | 0.80 |  |
| Male sex * Current smoking | 0.93 | 0.95 | 0.97 | 0.90 |  |
| Male sex * non-HDL cholesterol | 0.96 | 0.96 | 0.94 | 0.97 |  |
| Male sex * PAD | 1.02 | 1.02 | 0.95 | 1.08 |  |
| Male sex * SBP | 1.00 | 1.00 | 1.00 | 1.00 |  |
| Male sex * Years since first ASCVD diagnosis - square root term | 1.06 | 1.07 | 1.07 | 1.06 |  |
| Original hazard ratios were obtained by exponentiating coefficients from unpenalized Cox regression fitted to one randomly selected imputed dataset for computational reasons. For this reason, coefficients can differ from the penalized coefficients in the linear predictor in the model. Sensitivity analyses were performed on the same imputed dataset using unpenalized Cox regression. Sensitivity analysis I excluded events in the first 6 months after baseline. Sensitivity analysis II excluded HF death as incident HF event. Sensitivity analysis III censored patients with myocardial infarction events prior to their HF event. Abbreviations: HF = heart failure; MI = myocardial infarction; AAA = abdominal aortic aneurysm; BMI = body mass index; CeVD = cerebrovascular disease; CRP = C-reactive protein; eGFR = estimated glomerular filtration rate; HDL = high density lipoprotein; PAD = peripheral artery disease; SBP = systolic blood pressure; ASCVD = atherosclerotic cardiovascular disease. | | | | | |

**Supplementary Figures**

**Figure S1:** Flowchart of the study population in UCC-SMART cohort

Abbreviations: ASCVD = atherosclerotic cardiovascular disease; HF = heart failure.
Linkage to Statistics Netherlands was not possible in 945 patients, as linkage relied on a combination of birthdate, gender and postal code. Not all individuals in the UCC-SMART cohort could be uniquely linked using that combination.
No follow-up data were available for 642 patients included before 2013, as linkage to hospital data at that time also relied on a combination of birthdate, gender and postal code. As a result, these individuals could not be uniquely linked to hospital data at their time of inclusion in the UCC-SMART cohort. Inclusion of these patients could introduce immortal time bias, which is why these individuals were excluded.

**Figure S2.** European risk regions based on standardized CVD mortality rates

Risk regions based on most recently available age- and sex-standardized overall cardiovascular disease (CVD) mortality rates per 100,000: low risk (≤100 CVD deaths per 100,000), moderate risk (100 to <150 CVD deaths per 100,000), high risk (150 to <300 CVD deaths per 100,000), and very high risk ($\geq$300 CVD deaths per 100,000). Estimates are obtained from the WHO cause specific mortality database (2020).^18^

**Figure S3.** Smoothing of baseline survival probabilities

Presented plots show the 1-year baseline survivals across age for both outcome (red dots). These estimates where smoothed using a Nonlinear Restricted Splines function for both outcomes. Smoothed baseline survivals are presented by the blue line. The methodology is described in the Supplementary Methods section, and all baseline survivals after smoothing are provided in Table S7.

*Survival estimates (ages 40-90) for incident HF*

S*urvival estimates (ages 40-90) for non-HF death*

## Figure S4. Internal model performance in UCC-SMART

Calibration of incident HF and non-HF death in the derivation cohort (UCC-SMART), stratified for the complete population and women and men separately. Calibration plots show deciles of predicted risks plotted against observed cumulative incidence. Internal C-statistics are displayed inside the plot of the corresponding outcome and population.

## Figure S5. C-statistics in external validation stratified by sex

Discrimination in all external validation data is based on Harrell’s C-statistic. Discrimination was assessed at 10 years (CPRD, HUNT3, SWEDEHEART) or at the 75^th^ percentile of follow-up duration for ASCVD-Particles (9 years), Estonian Biobank (5 years) and REACH (2 or 3 years, depending on the region). Subgroups with less than 30 events were deemed too small to assess discrimination and are not shown in this plot. Abbreviations: EU = Europe; N = number of patients; CI = confidence interval.

**Figure S6.** C-statistics in external validation stratified by ASCVD type

Discrimination in all external validation data is based on Harrell’s C-statistic. Discrimination was assessed at 10 years (CPRD, HUNT3, SWEDEHEART) or at the 75^th^ percentile of follow-up duration for ASCVD-Particles (9 years), Estonian Biobank (5 years) and REACH (2 or 3 years, depending on the region). Subgroups with less than 30 events were deemed too small to assess discrimination and are not shown in this plot. Subgroups of patients with peripheral artery disease and abdominal aortic aneurysm were combined due to low prevalence in validation data sources. Abbreviations: CAD = coronary artery disease; CeVD = cerebrovascular disease; PAD = peripheral artery disease; AAA = abdominal aortic aneurysm; EU = Europe; CI = confidence interval

**Figure S7.** Calibration of incident HF in women

Calibration of incident HF for women in external validation data sources. Smoothed calibration plots of predicted risks versus observed risks and a histogram of the predicted risks (in blue) is shown. Calibration is shown at 10 years where possible, or at the 75th percentile of follow-up duration for ASCVD-Particles (9-year risks) and Estonian Biobank (5-year risks). Abbreviations: HF = heart failure.

**Figure S8.** Calibration of incident HF in men

Calibration of incident HF for men in external validation data sources. Smoothed calibration plots of predicted risks versus observed risks and a histogram of the predicted risks (in blue) is shown. Calibration is shown at 10 years where possible, or at the 75th percentile of follow-up duration for ASCVD-Particles (9-year risks) and Estonian Biobank (5-year risks). Abbreviations: HF = heart failure.

**Figure S9.** Calibration of non-HF mortality in external validation

Calibration of non-HF death in external validation data sources. Smoothed calibration plots of predicted risks versus observed risks and a histogram of the predicted risks (in blue) is shown. Calibration is shown at 10 years where possible, or at the 75th percentile of follow-up duration for ASCVD-Particles (9-year risks) and Estonian Biobank (5-year risks). Abbreviations: HF = heart failure.

## Figure S10. Decision curve analysis

Decision curve analysis in data sources with long-term follow-up (9 years for ASCVD-Particles and 10 years for the other data sources). Each panel displays the net benefit of treatment intensification based on the SMART2-HF model (blue) compared with the “treat-all” (red) and “treat-none” (green) strategies.

**Figure S11.** Comparison of external discrimination of alternative models in two external validation data sources (sensitivity analysis)

Discrimination (based on Harrell’s C- statistic) of the SMART2-HF model and two alternative model development approaches: sex-specific modelling (‘sex-specific derivation’) and model derivation in SWEDEHEART (‘SWEDEHEART derivation’) in external validation data from HUNT3 and ASCVD-Particles. Discrimination was assessed at 10-years (HUNT3) and 9-years (ASCVD-Particles) for the total population and for men and women separately. C-statistics were assessed prior to recalibration and can therefore slightly differ from those in Figure 1 and S5 for the SMART2-HF model. Abbreviations: CI = confidence interval.

## Figure S12. Distribution of SMART2-HF and SMART2 predicted risks

The figure displays the distribution of 10-year incident HF risks (predicted with SMART2-HF) and 10-year recurrent CV risks (predicted with SMART2) in the validation population of HUNT3 (low risk region). Dashed lines correspond with the sex-specific 25^th^, 50^th^ and 75^th^ percentiles of the respective risk distribution. Colours are used for illustrative purposes only and correspond with the colours used in Figure 3.

**Figure S13.** Estimation of individual risk of incident HF and individual treatment benefit

Estimation of individual 10-year and lifetime risk and individual treatment benefit. The benefit from lowering systolic blood pressure with 20 mmHg for two different ASCVD patients is shown. Treatment benefits were estimated by combining the SMART2-HF model with the hazard ratio (HR 0.72 per 10 mmHg SBP lowering for incident HF and HR 0.87 per 10 mmHg SBP lowering for non-HF death) from a meta-analysis.^15^ The individual treatment benefit can be shown as an absolute risk reduction (barchart, bottom row) or as gain in HF-free lifeyears (dark green area in survival plot, top row). Uncertainty for gain in HF-free lifeyears was determined by sampling (n=1000) from a log-normal distribution following the 95% CI from the meta-analysis.
Abbreviations: MI = myocardial infarction; SBP = systolic blood pressure; BMI = body mass index; non-HDL = high-density lipoprotein; eGFR = estimated glomerular filtration rate; CRP = C-reactive protein; CI = confidence interval. Created in <https://BioRender.com>.

**Supplementary References**

1.	Herrett E, Gallagher AM, Bhaskaran K, Forbes H, Mathur R, van Staa T, et al. Data Resource Profile: Clinical Practice Research Datalink (CPRD). Int J Epidemiol. 2015 Jun;44(3):827–36. doi:10.1093/ije/dyv098 PubMed PMID: 26050254.

2.	Krokstad S, Langhammer A, Hveem K, Holmen TL, Midthjell K, Stene TR, et al. Cohort Profile: the HUNT Study, Norway. Int J Epidemiol. 2013 Aug;42(4):968–77. doi:10.1093/ije/dys095 PubMed PMID: 22879362.

3.	Jernberg T, Attebring MF, Hambraeus K, Ivert T, James S, Jeppsson A, et al. The Swedish Web-system for enhancement and development of evidence-based care in heart disease evaluated according to recommended therapies (SWEDEHEART). Heart. 2010 Oct;96(20):1617–21. doi:10.1136/hrt.2010.198804 PubMed PMID: 20801780.

4.	Kuźma Ł, Dąbrowski EJ, Kurasz A, Święczkowski M, Jemielita P, Kowalewski M, et al. Effect of air pollution exposure on risk of acute coronary syndromes in Poland: a nationwide population-based study (EP-PARTICLES study). The Lancet regional health Europe. 2024 Jun;41:100910. doi:10.1016/j.lanepe.2024.100910 PubMed PMID: 38665621.

5.	Leitsalu L, Haller T, Esko T, Tammesoo ML, Alavere H, Snieder H, et al. Cohort Profile: Estonian Biobank of the Estonian Genome Center, University of Tartu. Int J Epidemiol. 2015 Aug;44(4):1137–47. doi:10.1093/ije/dyt268 PubMed PMID: 24518929.

6.	Ohman EM, Bhatt DL, Steg PG, Goto S, Hirsch AT, Liau CS, et al. The REduction of Atherothrombosis for Continued Health (REACH) Registry: an international, prospective, observational investigation in subjects at risk for atherothrombotic events-study design. Am Heart J. 2006 Apr;151(4):786.e1-10. doi:10.1016/j.ahj.2005.11.004 PubMed PMID: 16569533.

7.	Bhatt DL, Eagle KA, Ohman EM, Hirsch AT, Goto S, Mahoney EM, et al. Comparative determinants of 4-year cardiovascular event rates in stable outpatients at risk of or with atherothrombosis. JAMA. 2010 Sep 22;304(12):1350–7. doi:10.1001/jama.2010.1322 PubMed PMID: 20805624.

8.	Bhatt DL, Steg PG, Ohman EM, Hirsch AT, Ikeda Y, Mas JL, et al. International prevalence, recognition, and treatment of cardiovascular risk factors in outpatients with atherothrombosis. JAMA. 2006 Jan 11;295(2):180–9. doi:10.1001/jama.295.2.180 PubMed PMID: 16403930.

9.	Steg PG, Bhatt DL, Wilson PWF, D’Agostino R, Ohman EM, Röther J, et al. One-year cardiovascular event rates in outpatients with atherothrombosis. JAMA. 2007 Mar 21;297(11):1197–206. doi:10.1001/jama.297.11.1197 PubMed PMID: 17374814.

10.	Rubin DB. Multiple imputation for nonresponse in surveys. . New York: John Wiley & Sons; 1987.

11.	Hageman SHJ, McKay AJ, Ueda P, Gunn LH, Jernberg T, Hagström E, et al. Estimation of recurrent atherosclerotic cardiovascular event risk in patients with established cardiovascular disease: the updated SMART2 algorithm. Eur Heart J. 2022 May 7;43(18):1715–27. doi:10.1093/eurheartj/ehac056 PubMed PMID: 35165703.

12.	Kaasenbrood L, Bhatt DL, Dorresteijn JAN, Wilson PWF, D’Agostino RB, Massaro JM, et al. Estimated Life Expectancy Without Recurrent Cardiovascular Events in Patients With Vascular Disease: The SMART-REACH Model. J Am Heart Assoc. 2018 Aug 21;7(16):e009217. doi:10.1161/JAHA.118.009217 PubMed PMID: 30369323.

13.	Jaspers NEM, Blaha MJ, Matsushita K, van der Schouw YT, Wareham NJ, Khaw KT, et al. Prediction of individualized lifetime benefit from cholesterol lowering, blood pressure lowering, antithrombotic therapy, and smoking cessation in apparently healthy people. Eur Heart J. 2020 Mar 14;41(11):1190–9. doi:10.1093/eurheartj/ehz239 PubMed PMID: 31102402.

14.	Berkelmans GFN, Gudbjörnsdottir S, Visseren FLJ, Wild SH, Franzen S, Chalmers J, et al. Prediction of individual life-years gained without cardiovascular events from lipid, blood pressure, glucose, and aspirin treatment based on data of more than 500 000 patients with Type 2 diabetes mellitus. Eur Heart J. 2019 Sep 7;40(34):2899–906. doi:10.1093/eurheartj/ehy839 PubMed PMID: 30629157.

15.	Ettehad D, Emdin CA, Kiran A, Anderson SG, Callender T, Emberson J, et al. Blood pressure lowering for prevention of cardiovascular disease and death: a systematic review and meta-analysis. Lancet. 2016 Mar 5;387(10022):957–67. doi:10.1016/S0140-6736(15)01225-8 PubMed PMID: 26724178.

16.	Levey AS, Stevens LA, Schmid CH, Zhang YL, Castro AF, Feldman HI, et al. A new equation to estimate glomerular filtration rate. Ann Intern Med. 2009 May 5;150(9):604–12. doi:10.7326/0003-4819-150-9-200905050-00006 PubMed PMID: 19414839.

17.	Berkelmans GFN, Read SH, Gudbjörnsdottir S, Wild SH, Franzen S, van der Graaf Y, et al. Population median imputation was noninferior to complex approaches for imputing missing values in cardiovascular prediction models in clinical practice. J Clin Epidemiol. 2022 May;145:70–80. doi:10.1016/j.jclinepi.2022.01.011 PubMed PMID: 35066115.

18.	World Health Organization. WHO Mortality Database. [Internet]. [cited 2024 Dec 6]. Available from: https://platform.who.int/mortality
